# Biosynthesis of Largimycins in *Streptomyces argillaceus* Involves Transient β-Alkylation and Cryptic Halogenation Steps Unprecedented in the Leinamycin Family

**DOI:** 10.1021/acschembio.2c00416

**Published:** 2022-07-13

**Authors:** Adriana Becerril, Ignacio Pérez-Victoria, Jesús M. Martín, Fernando Reyes, Jose A. Salas, Carmen Méndez

**Affiliations:** † Departamento de Biología Funcional e Instituto Universitario de Oncología del Principado de Asturias (I.U.O.P.A), Universidad de Oviedo, 33006 Oviedo, Spain; ‡ Instituto de Investigación Sanitaria de Asturias (ISPA), 33011 Oviedo, Spain; § Fundación MEDINA, Centro de Excelencia en Investigación de Medicamentos Innovadores en Andalucía, Armilla, 18016 Granada, Spain

## Abstract

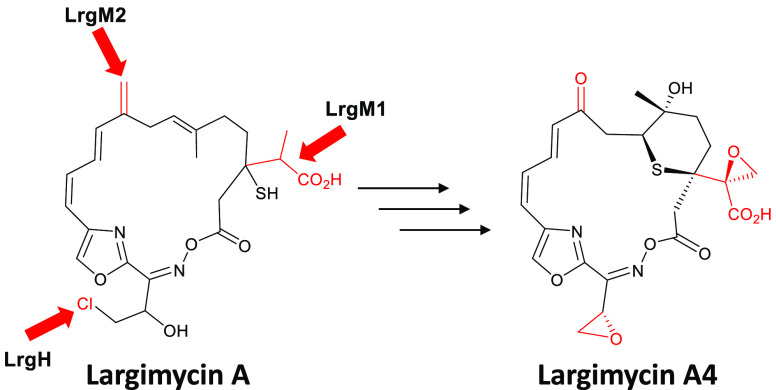

Largimycins A1 and A2 are key members of a recently identified family of hybrid nonribosomal peptide polyketides belonging to the scarcely represented group of antitumor leinamycins. They are encoded by the gene cluster *lrg* of *Streptomyces argillaceus*. This cluster contains a halogenase gene and two sets of genes for the biosynthesis and incorporation of β branches at C3 and C9. Noticeably, largimycins A1 and A2 are nonhalogenated compounds and only contain a β branch at C3. By generating mutants in those genes and characterizing chemically their accumulated compounds, we could confirm the existence of a chlorination step at C19, the introduction of an acetyl-derived olefinic exomethylene group at C9, and a propionyl-derived β branch at C3 in the biosynthesis pathway. Since the olefinic exomethylene group and the chlorine atom are absent in the final products, those biosynthetic steps can be considered cryptic in the overall pathway but essential to generating keto and epoxide functionalities at C9 and C18/C19, respectively. We propose that chlorination at C19 is utilized as an activation strategy that creates the precursor halohydrin to finally yield the epoxy functionality at C18/C19. This represents a novel strategy to create such functionalities and extends the small number of natural product biosynthetic pathways that include a cryptic chlorination step.

Polyketides (PKs) are a major class of natural products that show important activities such as antibiotic, antitumor, or immunosuppressant ones. They are synthesized by polyketide synthases (PKSs) through sequential decarboxylative condensations of acyl-CoA units.^1^ AT-less (or *trans*-) PKSs are type I PKSs characterized by lacking AT domains, which are provided *in trans* by discrete proteins at each elongation step.^2,3^ This type of PKS shows some special features such as modules splitting on two proteins, domains acting across modules, or functions provided *in trans*.^3^ Quite often PKs synthesized by AT-less PKS contain β branches, which usually are incorporated by hydroxymethylglutaryl-CoA synthases (HMGS) that catalyze condensation of an acyl-ACP to a β-keto group of the ACP-tethered PK growing chain to generate a hydroxyacyl-ACP intermediate.^4,5^ This biosynthesis step involves a set of discrete proteins/domains that together with HMGS are known as “HMG” or “HCS-cassettes”.^4,5^ These usually include two or three discrete proteins for generating the acyl-ACP from acyl-CoA (a donor ACP, an AT, and a KS that lacks a conserved cysteine residue and acts as a decarboxylating enzyme) and one or two enzymes homologous to enoyl-CoA hydratase (ECH) superfamily members, which dehydrate (ECH1) and decarboxylate (ECH2) the hydroxyacyl-ACP intermediate and that can exist as discrete proteins or as domains within the PKS.^4−7^

*Streptomyces argillaceus* is a well-known producer of antitumor mithramycin.^8^ Moreover, it has the potential to produce at least 30 more specialized metabolites, encoded by additional biosynthesis gene clusters (BGCs), as it has been uncovered by genome mining.^9^ Most of these BGCs are silent or low expressed under standard laboratory conditions. In recent years, expression of some of them has been activated/increased and their encoded compounds identified.^9,10^ Very recently also, the cryptic BGC *lrg* (MIBIG accession number BGC0001853) has been identified and activated, and the encoded compounds named largimycins (LRG) A1 and LRG A2 have been identified (Figure 1).^11^ LRGs are hybrid peptide-polyketide compounds that constitute a new group within the unusual leinamycin (LNM) family of natural products.^11^ They show unique structural features that differentiate them from the three known groups of this family of compounds identified so far.^12^ Thus, LRGs contain a 19-membered instead of an 18-membered macrocyclic ring; they are cyclized through an oxime ester, an unprecedented structural feature in natural products, and they have an oxazole instead of a thiazole ring (Figure 1a). The *lrg* BGC contains several genes encoding putative proteins for the incorporation of β branches to the polyketide growing chain: *lrgM1* and *lrgM2* for HMGS homologues, *lrgK* for a bifunctional AT/DC, *lrgL* and *lrgA* for discrete ACPs, *lrgD* for a discrete decarboxylating KS, and *lrgF* for enoyl-CoA hydratase (ECH1; Figure 1b). In addition, module 5 of LrgJ PKS contains an Enoyl-CoA hydratase (ECH2) domain, and module 8, two tandem ACP domains (Figure 1b). Tandemly duplicated ACP domains are found in modules involved in β-alkylation.^13,14^ The presence of these proteins and the PKS domain organization suggests that during LRG biosynthesis two β branches are incorporated at the PK growing chain at C9 and C3. Accordingly, LRG A1 and LRG A2 contain a side chain at C3, but noticeably they lack any at C9 (Figure 1a).^11^ Interestingly, the biosynthesis intermediate LRG O1 produced by mutant *S. argillaceus* ΔlrgO that is affected in the FAD-dependent oxidoreductase LrgO contains an olefinic exomethylene group at C-9 (Figure 1a).^11^ On the other hand, the *lrg* BGC contains a nom-heme iron dependent halogenase *lrgH* coding gene, but noticeably LRG A1 and LRG A2 are nonhalogenated compounds (Figure 1a).

**Figure 1:**
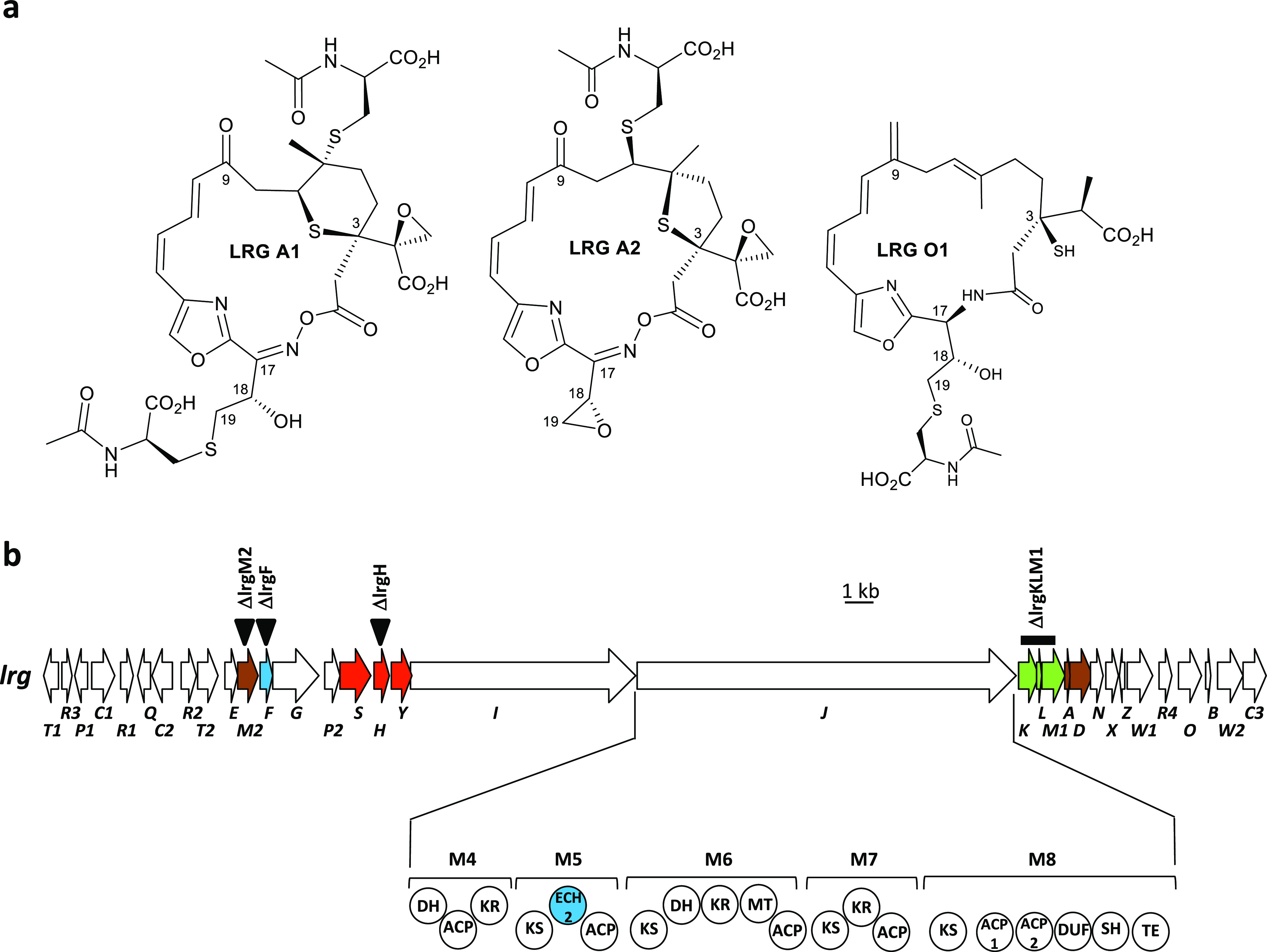
Largimycins. (a) Chemical structures of largimycin (LRG) A1, LRG A2, and LRG O1. (B) Genetic organization of *lrg* BGC from *Streptomyces argillaceus*. Largimycins genes (*lrg*) involved in β branching at C3 and at C9 are in green and brown, respectively. The ECH1 coding gene and the ECH2 domain are shown in blue. Genes encoding the loading module of NRPS are in red. Other *lrg* genes are shown in white. Organization of domains within modules in LrgJ PKS is shown. Black triangles and bars indicate those genes that have been inactivated or deleted. Genes are shown to scale.

Here, we report the identification of genes responsible for the installation of a propionyl- and of an acetyl-derived β-alkyl group at C3 and C9, respectively, in the PK growing chain during LRG biosynthesis, and we also show that alkylation at C9 is cryptic in the overall pathway for C9 keto group formation. We also determine the existence of a cryptic halogenation step catalyzed by halogenase LrgH, which is transient in the overall pathway to epoxide group formation at C18/C19.

## Results and Discussion

### Deletion of *lrgKLM1* Leads to the Accumulation of Largimycin Intermediates Lacking the β Branch at C-3

**Figure 2:**
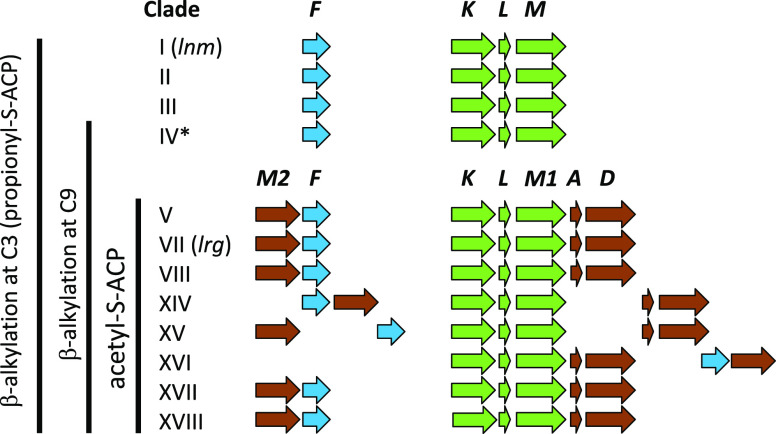
Genetic organization of β branching genes in *lnm*-type gene clusters. Comparison of clades proposed to encode compounds with β-alkyl branches at C3 using propionyl-*S*-ACP as a substrate.^12^ Clades IV, V, VII, VIII, and XIV to XVIII would encode compounds with additional β branches at C9, either derived from propionyl-*S*-ACP (clade IV) or from acetyl-*S*-ACP. Genes shown are named as in *lnm* BGC^39^ and/or as in *lrg* BGC.^11^ Genes for β branching at C3 and at C9 are in green and brown, respectively. Genes coding for ECH1 are shown in blue. Information to generate this figure was extracted from references (11 and 12).

**Figure 3:**
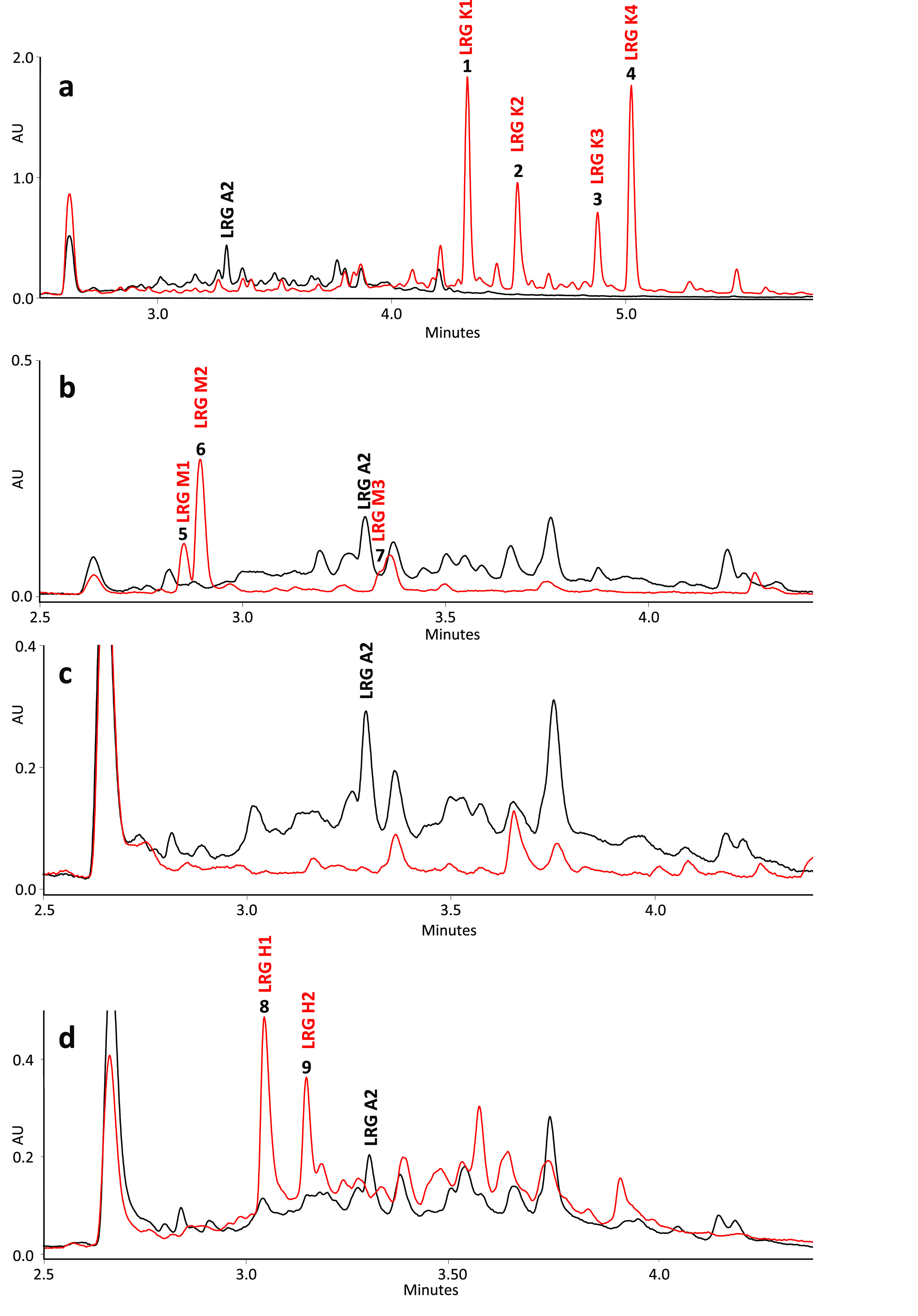
Production of largimycins by *S. argillaceus* mutants. UPLC chromatograms of ethyl acetate extracts of *S. argillaceus* WT-R2 (black line) in comparison to mutant strains (red line): *S. argillaceus* ΔlrgKLM1-R2 obtained at 300 nm (a); *S. argillaceus* ΔlrgM2-R2 obtained at 365 nm (b); *S. argillaceus* ΔlrgF-R2 obtained at 330 nm (c); and *S. argillaceus* ΔlrgH-R2 obtained at 330 nm (d). Peaks with numbers correspond to compounds produced by the mutants that have been purified for chemical characterization. The peak for LRG A2 is indicated and labeled in black.

**Figure 4:**
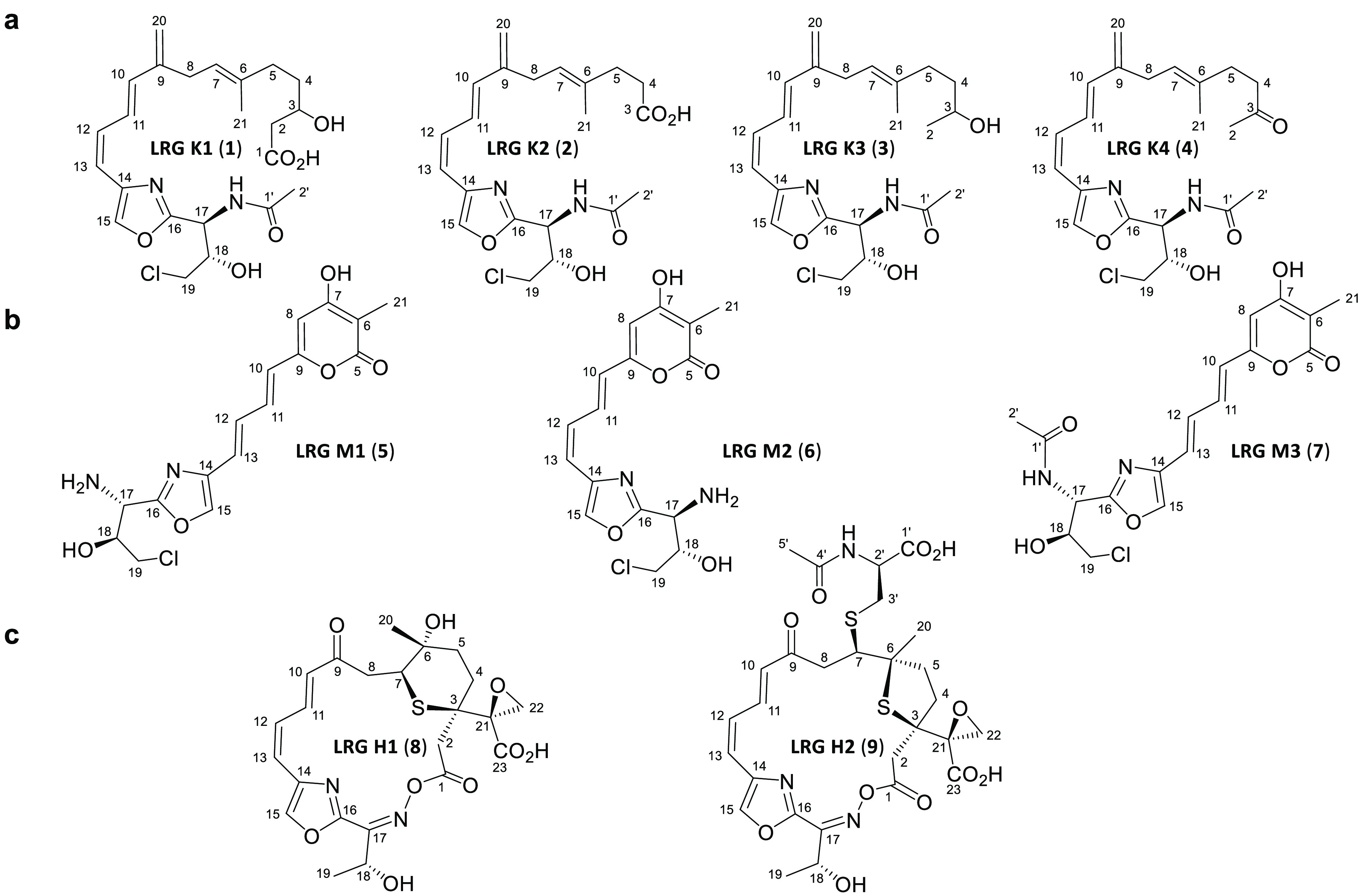
Chemical structures of largimycins (LRGs) produced by *S. argillaceus* ΔlrgKLM1-R2 (a); *S. argillaceus* ΔlrgM2-R2 (b); and *S. argillaceus* ΔlrgH-R2 (c).

**Figure 5:**
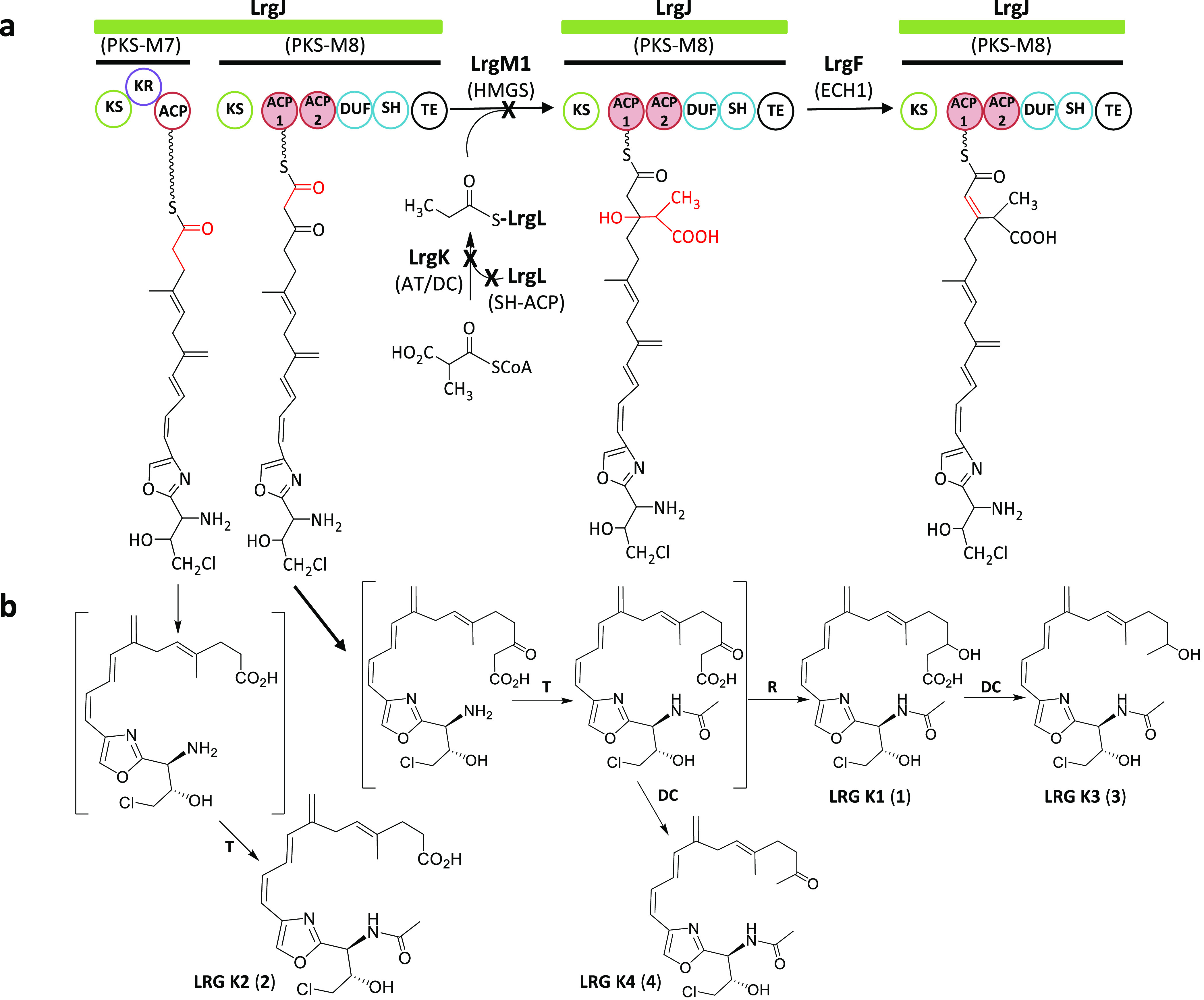
Proposed biosynthetic pathway for β-alkylation at C3 in largimycins. Biosynthetic pathway in the wild type strain (a). Formation of accumulated compounds in *S. argillaceus* ΔlrgKLM1 (b). DC, decarboxylase; R, reductase; T, acetyl transferase.

The *lrg* BGC contains two HMGS coding genes, *lrgM1* and *lrgM2* (Figure 1b). *LrgM1* is clustered together with *lrgK* and *lrgL* downstream of the AT-less PKS *lrgJ*. These three genes show homology to *lnmKLM* from the leinamycin BGC (*lnm*), which have been shown to be involved in the installation of the β branched C3 unit in LNM.^7,15^ Homologues to those genes and showing the same genetic organization are found in all *lnm*-type BGCs identified so far, which encode putative compounds with propionyl-derived β-alkyl branches at C3 (Figure 2).^12^ To determine the role of *lrgKLM1* in LRG biosynthesis, those genes were jointly deleted from *S. argillaceus* using pHZ-orf39–41 (Table 1). The genotype of the resultant mutant strain *S. argillaceus* ΔlrgKLM1 was confirmed by PCR (Figure S1). After expressing the cluster specific activator *lrgR2* into it, the resultant recombinant strain *S. argillaceus* ΔlrgKLM1-R2 (Table 1) was analyzed for LRG production. UPLC analyses of organic extracts showed that LRG production was abolished in *S. argillaceus* ΔlrgKLM1-R2, confirming that these three genes were essential for biosynthesis of LRG. Instead, this mutant produced four major compounds (**1** to **4**, Figure 3a), which were purified and chemically characterized by MS and NMR (see Supporting Information). They correspond to new compounds that were named LRG K1 (**1**), LRG K2 (**2**), LRG K3 (**3**), and LRG K4 (**4**) (Figure 4a). All of these compounds contain a hybrid peptide-polyketide chain N-acetylated at the threonine residue and lack a β branch at C3 that confirms the involvement of *lrgKLM1* in the formation and installation of this alkyl group. Remarkably, all compounds contain an olefinic β-exomethylene group at C9. Incorporation of this group has been hypothesized to occur during LRG biosynthesis, even though the final products lack it (Figure 1a).^11^ The presence of that group in LRG K1–K4 confirms that biosynthesis of LRG proceeds through intermediates containing the olefinic β-exomethylene group (Figure 5) and indicates that *lrgKLM1* is not involved in that process. Noticeably, all of these compounds are also chlorinated at C19. Although LRGs lack chlorine, a halogenation step was suggested to occur along the biosynthesis pathway based on the identification of the halogenase gene *lrgH* in the BGC.^11^ Detection of chlorine in compounds LRG K1–K4 corroborates that a halogenation step occurs during LRG biosynthesis.

**Table 1:** *Streptomyces argillaceus* Strains and Plasmids Generated in This Work

mutant strain	inactivated gene(s)	plasmid
*S. argillaceus* ΔlrgKLM1	*lrgK, lrgL, lrgM1*	pHZ-orf39–41
*S. argillaceus* ΔlrgM2	*lrgM2*	pHZ-HMG
*S. argillaceus* ΔlrgF	*lrgF*	pHZ-LrgF
*S. argillaceus* ΔlrgKLM1ΔlrgH	*lrgK, lrgL, lrgM1, lrgH*	pHZ-orf39–41
		pHZ-HalSyrB2
*S. argillaceus* ΔlrgH	*lrgH*	pHZ-HalSyrB2

recombinant strain	expressed gene(s)	plasmid
*S. argillaceus* ΔlrgKLM1-R2	*lrgR2*	pEM4T-R2
*S. argillaceus* ΔlrgKLM1-R2-KLM1	*lrgR2*	pEM4T-R2
	*lrgKLM1*	pSETEH-orf39–41
*S. argillaceus* ΔlrgM2-R2	*lrgR2*	pEM4T-R2
*S. argillaceus* ΔlrgM2-R2-M2	*lrgR2*	pEM4T-R2
	*lrgM2*	pSETEH-HMG
*S. argillaceus* ΔlrgM2-R2-EM2	*lrgR2*	pEM4T-R2
	*lrgE, lrgM2*	pSETEH-LrgE-HMG
*S. argillaceus* ΔlrgF-R2	*lrgR2*	pEM4T-R2
*S. argillaceus* ΔlrgF-R2-F	*lrgR2*	pEM4T-R2
	*lrgF*	pSETEH-LrgF
*S. argillaceus* ΔlrgKLM1ΔlrgH-R2	*lrgR2*	pEM4T-R2
*S. argillaceus* ΔlrgH-R2	*lrgR2*	pEM4T-R2
*S. argillaceus* ΔlrgH-R2-H	*lrgR2*	pEM4T-R2
	*lrgH*	pSETec-H

### Mutants in *lrgM2* Accumulate Truncated Intermediates Lacking the Olefinic β-Exomethylene Group

**Figure 6:**
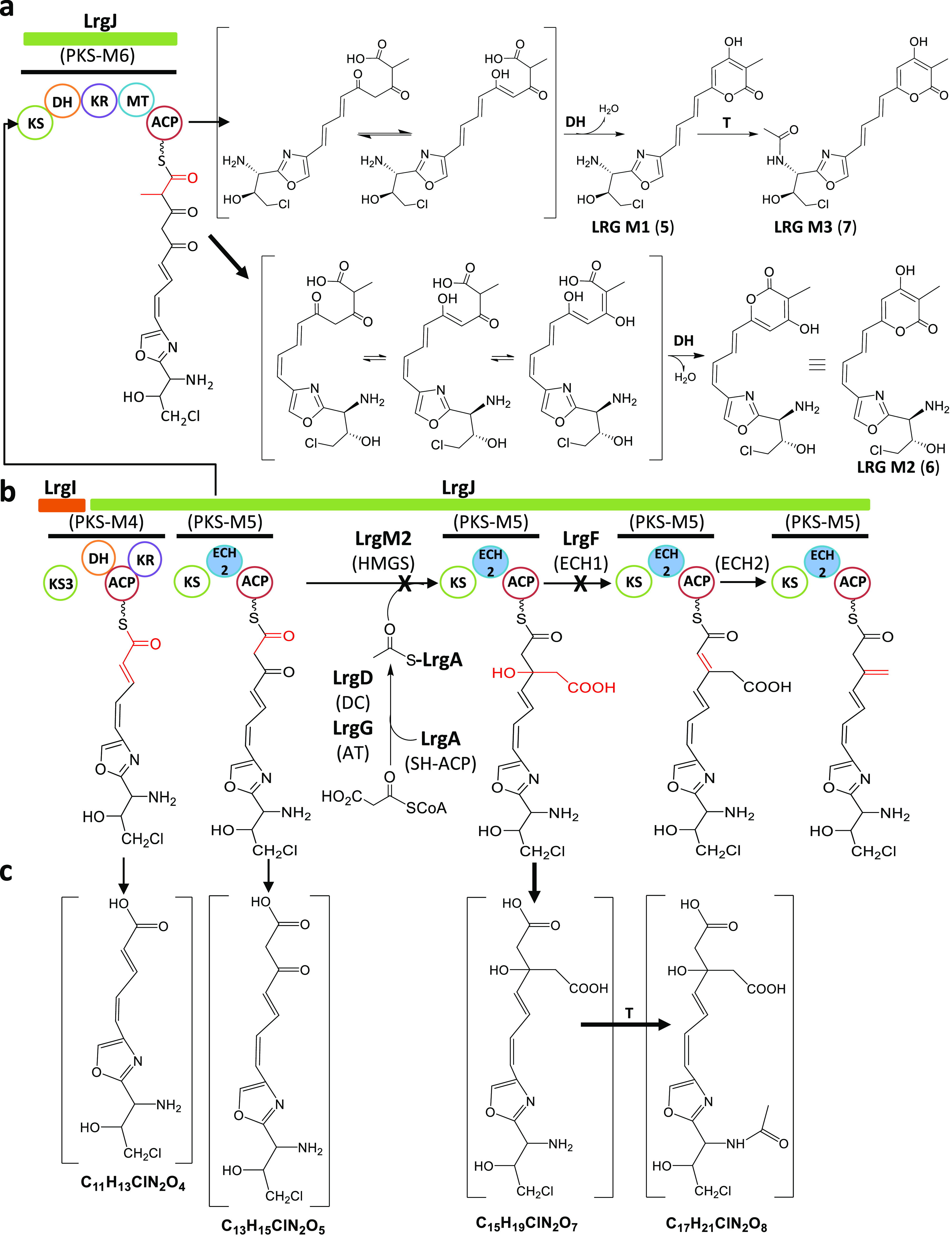
Proposed biosynthetic pathway for the formation of the olefinic β-exomethylene group during largimycins biosynthesis. Biosynthesis of the olefinic β-exomethylene group during largimycins biosynthesis in the wild type strain (b). Formation of compounds accumulated by *S. argillaceus* ΔlrgM2 (a) and by *S. argillaceus* ΔlrgF (c). DH, dehydratase; T, acetyl transferase.

The second HMGS coding gene *lrgM2* is located upstream of the hybrid NRPS/AT-less type I PKS encoding gene *lrgI* (Figure 1b). To deepen into its role in LRG biosynthesis, *lrgM2* was independently inactivated using pHZ-HMG (Table 1). The resultant mutant *S. argillaceus* ΔlrgM2 was genetically confirmed by PCR (Figure S1). After expressing *lrgR2* into that mutant, the resultant recombinant strain *S. argillaceus* ΔlrgM2-R2 (Table 1) was cultivated, and its metabolite profile was analyzed by UPLC. As observed in Figure 3b, this mutant was blocked in LRG production, confirming that LrgM2 is required for LRG biosynthesis, but it accumulated several compounds instead (**5** to **7**; Figure 3b). Purification and structural characterization of these compounds (see Supporting Information) revealed that they were new compounds that were named LRG M1 (**5**), LRG M2 (**6**), and LRG M3 (**7**; Figure 4b). All of them contain a truncated peptide-polyketide chain that lacks the olefinic β-exomethylene group at C9. This confirms the role of LrgM2 in incorporating this β branch at C9 during LRG biosynthesis (Figure 6). Since the final products of the LRG pathway (LRG A1 and LRG A2) lack that group at C9, it was initially thought that the biosynthesis of LRGs could follow two alternative pathways: one involving a β-alkylation event at C9 and another avoiding this step, leading to compounds with an olefinic exomethylene or a keto group at C9, respectively. However, the phenotype of *S. argillaceus* ΔlrgM2 indicates that alkylation at C9 is mandatory for the LRG biosynthesis pathway to proceed, confirming that there is only one pathway for the biosynthesis of the LRG peptide-polyketide chain. Moreover, LRG M1–M3 also contain chlorine, reaffirming the existence of a halogenation step during LRG biosynthesis. Complementation of *S. argillaceus* ΔlrgM2-R2 was attempted by expressing *lrgM2 in trans* (pSETEH-HMG, Table 1), but it was not achieved. Curiously, when *lrgM2* was coexpressed with the upstream gene *lrgE* using pSETEH-LrgE-HMG (Table 1), production of LRGs was recovered (Figure S2). This suggests that *lrgE* and *lrgM2* are translationally coupled and should be cotranscribed from the same construct. A similar situation has been reported in the mupiricin BGC from *Pseudomonas fluorescens*, where some mutants in single genes could not be complemented *in trans* by the corresponding gene.^16^ They noticed that this happened when those genes were grouped as pairs, and complementation was only achieved when both genes were coexpressed together, independently if the mutated gene was the upstream or the downstream gene within a pair.

### Inactivation of *lrgF* Abolishes Largimycin Production

Downstream of *lrgM2* is located *lrgF* that encodes an enoyl-CoA hydratase homologue (ECH1). This is the only ECH1 encoding gene within the *lrg* BGC, and therefore it should be involved in dehydration steps during β branch formation at C3 and C9. To deepen into its role, *lrgF* was inactivated using plasmid pHZ-LrgF (Table 1), generating the mutant strain *S. argillaceus* ΔlrgF that was genetically confirmed by PCR (Figure S1). After expressing *lrgR2*, cultivation of the recombinant strain *S. argillaceus* ΔlrgF-R2 showed that production of LRGs was abolished, and no compound was accumulated in detectable amounts (Figure 3c). Since results mentioned above indicated that LRG biosynthesis proceeds through halogenated biosynthesis intermediates, we hypothesized that the *lrgF-*minus mutant would accumulate also halogenated compounds. Therefore, we analyzed cultures from this mutant looking for halogenated compounds. We could detect four compounds that were accumulated by *S. argillaceus* ΔlrgF-R2 in comparison to *S. argillaceus* WT-R2 (Figure S3). These compounds were present in minor amounts, but their molecular formulas could be assigned on the basis of experimental accurate masses observed in the LC-HRMS analysis, rendering C_15_H_19_ClN_2_O_7_, C_11_H_13_ClN_2_O_4_, C_17_H_21_ClN_2_O_8_, and C_13_H_15_ClN_2_O_5_. These molecular formulas are consistent with putative truncated chlorinated compounds released from modules 4 or 5 of the AT-less PKS LrgJ lacking the olefinic β-exomethylene group at C9 (compounds C_11_H_13_ClN_2_O_4_ and C_13_H_15_ClN_2_O_5_) or containing a nondehydrated, nondecarboxylated branch chain (C_15_H_19_ClN_2_O_7_) and its acetylated version (C_17_H_21_ClN_2_O_8_; Figure 6c). These results agree with the proposed function for LrgF as an ECH1 involved in the dehydration event leading to the formation of the alkyl branch at C9 (and most probably also at C3).

### Chlorination of Threonine Is Cryptic in the Overall Pathway of Epoxide Group Formation at C18/C19

**Figure 7:**
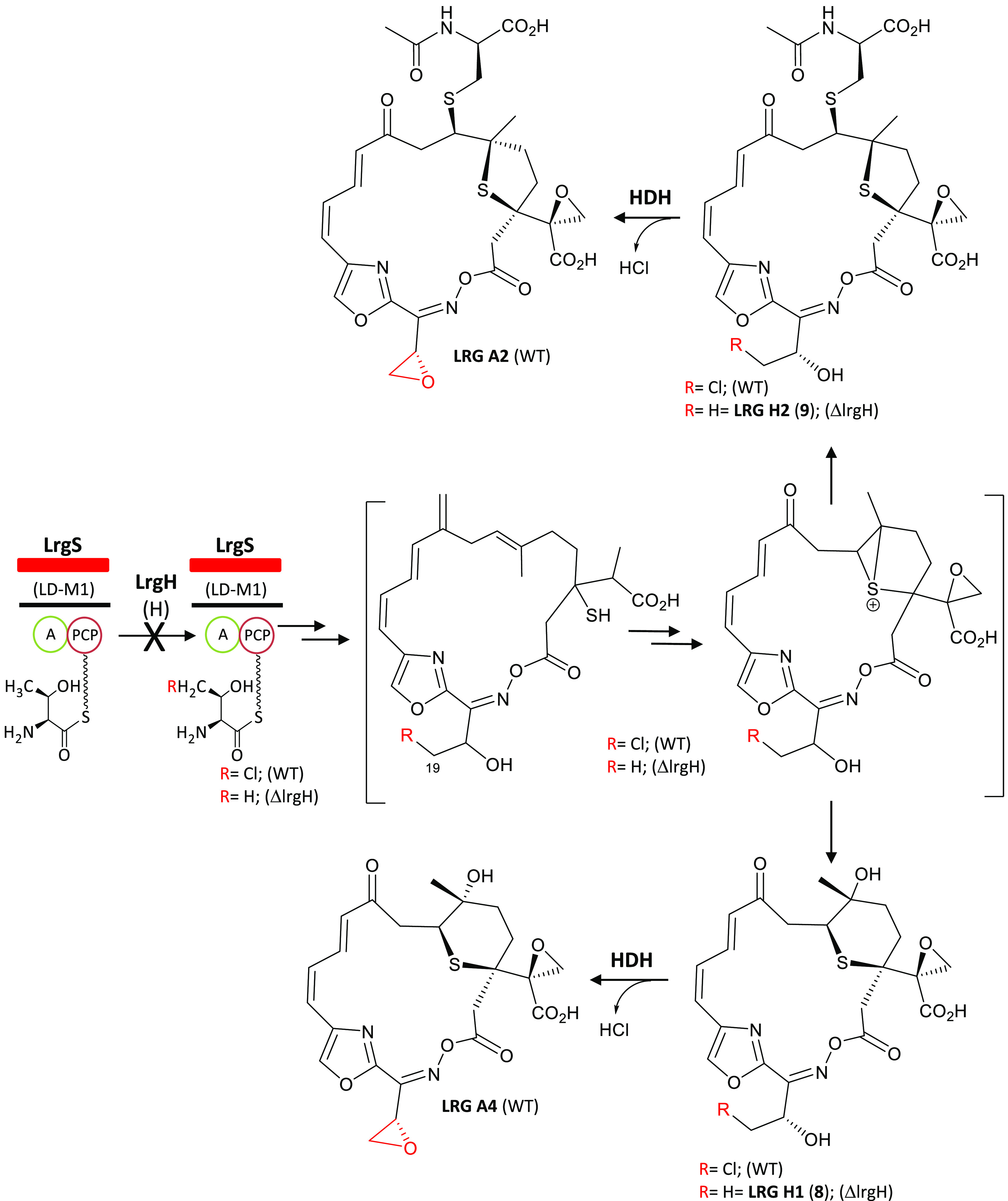
Proposed biosynthetic origin of LRG H1 and LRG H2. Biosynthesis of LRG A2 and LRG A4 in *S. argillaceus* wild type strain (WT) including the halogenation step by LrgH and biosynthesis of LRG H1 and LRG H2 in *S. argillaceus* ΔlrgH. H, halogenase; HDH, halohydrin dehalogenase.

The loading module for the hybrid NRPS/AT-less Type I PKS LrgI is constituted by three discrete proteins (LrgS, LrgH, and LrgY), which would activate and load l-threonine, halogenate it, and transfer the halogenated aminoacyl residue to LrgI, respectively.^11^ Since the final products LRG A1 and LRG A2 of the LRG biosynthesis pathway are nonhalogenated compounds, the existence of this halogenation step was uncertain. However, the identification of chlorinated biosynthesis intermediates (see above) confirms that LRG biosynthesis proceeds through chlorinated intermediates. To determine the role of LrgH in this halogenation step, a double mutant was generated by inactivating *lrgH* in the *S. argillaceus* ΔlrgKLM1 mutant using pHZ-HalSyrB2 (Table 1; Figure S1). Comparison of metabolite profiles from *S. argillaceus* ΔlrgKLM1-R2 and *S. argillaceus* ΔlrgKLM1ΔlrgH-R2 showed that production of LRGs K1–K4 was abolished in the latter strain, and interestingly, new metabolites consistent with nonchlorinated analogues of LRGs K1–K4 could be detected by LC/HRMS and ion extraction (Figure S4). These compounds showed masses and deduced molecular formulas corresponding to dechloro-LRG K1 (C_23_H_32_N_2_O_6_), dechloro-LRG K2 (C_21_H_28_N_2_O_5_), dechloro-LRG K3 (C_22_H_32_N_2_O_4_), and dechloro-LRG K4 (C_22_H_30_N_2_O_4_). These results suggest that *lrgH* encodes the halogenase responsible for introducing chlorine in LRGs K1–K4. They also indicate that biosynthesis of the hybrid peptide-polyketide chain can proceed in the absence of chlorination of l-Thr. To deepen into the role of LrgH in LRG biosynthesis, its coding gene was individually inactivated in the wild type strain using pHZ-HalSyrB2 (Table 1), generating *S. argillaceus* ΔlrgH (Figure S1). Organic extracts from *S. argillaceus* ΔlrgH-R2 showed that production of LRG A1 and LRG A2 was blocked, and several new metabolites were accumulated (Figure 3d). The two major compounds (**8** and **9** in Figure 3d) were purified and chemically characterized. They were new compounds named LRG H1 (**8**) and LRG H2 (**9**; Figure 4c). Their structures were very similar to those of LRG A2 (Figure 1a) and LRG A4^11^ but differing from them in the replacement of the epoxide group at C18/C19 of the side chain of LRG A2 and LRG A4 by a single hydroxy group at C18 in LRG H1 and LRG H2, thus displaying the standard Thr side chain. These results show that the LRG biosynthesis pathway can also proceed in the absence of the halogenation step and suggest that chlorination of the threonyl residue to render a halohydrin is required as an activation strategy for the formation of this epoxy group. Cryptic chlorinations have been described as events required prior to the formation of cyclopropyl rings and, in recent years, to the formation of terminal alkynes, ether and biaryl connections, and to other types of C–C rearrangements.^17,18^ The formation of an epoxide group in a putative biosynthesis intermediate of naphterpin and the marinone biosynthesis pathway has also been suggested, which would be generated from a dichlorinated substrate.^19^ Here, we propose that during LRG biosynthesis a cryptic chlorination event would take place en route to the formation of the epoxide group at C18/19 (Figure 7). This represents a novel strategy to create such functionalities and extends the small number of natural product biosynthetic pathways that include a cryptic chlorination step.^17^

According to results shown here and the bioinformatic analysis of *lrg* BGC, the biosynthesis and incorporation of the β branches at C3 and C9 during LRGs biosynthesis would involve two sets of HMGS cassettes and would proceed as follows (Figures 5 and 6). The β branch at C9 would derive from malonyl-CoA (Figure 6b). In a similar manner to what occurs in the biosynthesis of other malonyl-CoA derived β branches,^5,20^ the first step would be the transfer of malonyl from malonyl-CoA to the free-standing LrgA ACP. The LrgG *trans*-AT would catalyze this transfer, since this is the only AT for malonyl-CoA identified within the *lrg* BGC. LrgG would also be involved in loading the ACPs located at the different modules of the hybrid NRPS-PKS LrgI and the PKS LrgJ. Then, the KS LrgD would decarboxylate malonyl-*S*-LrgA to generate acetyl-*S*-LrgA. Next, the HMGS LrgM2 would perform the condensation of acetyl-*S*-LrgA with the ACP-tethered peptide-polyketide intermediate at module 5 of LrgJ PKS to generate an HMG-ACP thioester. Further dehydration by the ECH1 LrgF and decarboxylation by the ECH2 domain of LrgJ module 5 would lead to the formation of the olefinic β-exomethylene group located at C9 of the peptide-polyketide chain of LRGs. On the other hand, the β branch at C3 would derive from methylmalonyl-CoA (Figure 5a). In analogy to the alkylation process proposed in LNM biosynthesis,^7,15^ the AC/DC LrgK would catalyze both the transfer of methylmalonyl from methylmalonyl-CoA to the ACP LrgL and its subsequent decarboxylation to generate propionyl-*S*-LrgL. Then, the HMGS LrgM1 would condense the propionyl group to the peptide-polyketide chain attached to an ACP domain at module 8 of PKS LrgJ, followed by its dehydration by the ECH1 LrgF.

As mentioned above, blockage of the β-alkylation pathway either at C3 or at C9 interrupts LRG biosynthesis, and the stalled hybrid peptide-polyketide intermediates will be hydrolytically released from the PKS LrgJ. In the LNM or in the mupiricin biosynthesis pathways, mutations of genes involved in β-propionyl or β-methyl incorporation also lead to the formation of shortened compounds.^15,20−22^ Most probably, in the case of LRG, the release of compounds from the corresponding PKS modules will be performed by the type II TE LrgN,^11^ since this type of enzyme plays an editing role by eliminating abnormal growing chains from PKSs.^23,24^ Thus, in *S. argillaceus* ΔlrgM2, the hydrolytic release of a peptide-polyketide chain with an unreduced keto-group at the beta position of the end carboxylic acid from module 6 of PKS LrgJ would lead to the formation of LRG M2 and LRG M1 after a dehydration step, and to LRG M3 after further acetylation of the latter (Figure 6a). Also, in *S. argillaceus*, ΔlrgKLM1 compounds LRG K1, LRG K3, and LRG K4 would originate from the hydrolytic release of the chain attached intermediates at module 8 followed by their N-acetylation and either further decarboxylation (LRG K4) or reduction (LRG K1) and decarboxylation (LRG K3; Figure 5b). LRG K2 could result from the hydrolytic release of the peptide-polyketide chain intermediate from module 7, followed by its acetylation (Figure 5b). In the case of the putative compounds accumulated by *S. argillaceus* ΔlrgF (Figure 6c), the release of the alkylated peptide-polyketide chain from module 5 would originate compound C_13_H_19_ClN_2_O_7_ and its further acetylation compound C_17_H_21_ClN_2_O_8_. The hydrolytic release of truncated intermediates from module 5 before the alkylation event or from module 4 would lead to compounds C_13_H_15_ClN_2_O_5_ and C_11_H_13_ClN_2_O_4_, respectively.

Results shown in this work highlight the existence of two cryptic events during LRG biosynthesis, formation of an olefinic exomethylene group at C9 and chlorination of the threonyl residue. The olefinic exomethylene is finally converted into a keto group, probably after the peptide–polyketide chain is released from PKS LrgJ since the exomethylene group remains in all truncated biosynthesis intermediates identified so far, and in LRG O1 (Figure 1a). Possible candidates to carry out this transformation are cytochromes P450, which have been involved in this type of reaction in other pathways.^25^ The *lrg* gene cluster contains three cytochrome P450 encoding genes (*lrgC1*, *lrgC2*, and *lrgC3*), two of which (*lrgC1* and *lrgC3*) have been shown to be essential for LRG biosynthesis.^11^ Further studies would be required to determine if one of those genes is responsible for that modification. Candidates from the *lrg* BGC to remove the halogen from the threonyl residue with the concomitant formation of the epoxide group at C18/19 are not clear. Such transformation formally corresponds to halohydrin dehalogenase enzymatic activity, although halohydrin dehalogenases known so far are restricted to evolved bacterial enzymes capable of degrading halogenated xenobiotics from the postindustrial era.^26^ As expected, the *S. argillaceus* genome has no homologue of such halohydrin dehalogenases. Thus, the formation of the epoxide ring from the chlorinated Thr precursor in LRG biosynthesis represents an unprecedented type of halohydrin dehalogenase activity in secondary metabolism. Several types of enzymes have been described in natural products biosynthesis that carry out biochemical reactions involving the removal of halogens.^17^ These include Zn-dependent enzymes belonging to the vicinal oxygen chelate family of enzymes, FAD-dependent enzymes, and nicotinamide-dependent enzymes, for cyclopropanation reactions; PLP-dependent enzymes for terminal alkyne formation; or hemolysin-type calcium-binding protein for C-/O-alkylation reactions. The *lrg* gene cluster codes for several proteins of unknown function, including LrgB that contains a vicinal oxygen chelate domain. Further characterization of these genes will shed light on their roles in LRG biosynthesis and will clarify the enzymes involved in this novel halohydrin dehalogenase-like epoxidation reaction.

## Methods

### Strains, Culture Conditions, Plasmids, and DNA Manipulations

*S. argillaceus* ATCC 12956 and *S. argillaceus* WT-R2 were used as source of DNA and for LRGs production, respectively.^11^ LRG production analyses were performed using an SM30a medium.^11^ When required, media were supplemented with antibiotics at appropriate concentrations. DNA manipulations and transformation/conjugation of strains were carried out following standard procedures for *Streptomyces* and *E. coli*.^27,28^ Primers for PCR amplifications are listed in Table S1. Several plasmids were used: pUO9090 (M. C. Martín, unpublished results), pUK21,^29^ pLHyg,^30^ pHZ1358,^31^ pSETEH (R. García-Salcedo, unpublished results), pSETec,^32^ pEM4T,^33^ and pEM4T-R2.^11^

### Generation and Characterization of Mutant Strains

Several mutants were generated (Table 1 and Figure S1) by replacing most of the target gene by an apramycin resistance cassette that was inserted in the same direction of transcription to avoid polar effects on downstream genes. In the case of *S. argillaceus* ΔlrgH and in the double mutant *S. argillaceus* ΔlrgKLM1ΔlrgH, *lrgH* was replaced by a hygromycin resistance cassette. Mutants were genetically confirmed by PCR amplification and by sequencing the PCR products, using specific primers (Table S1). To confirm that mutations did not affect any other gene, mutant strains were complemented by expressing the corresponding gene(s) *in trans* (see below). To generate the mutants, the following plasmids were constructed as follows (Table 1). pHZ-orf39–41: A 1.94 kb DNA fragment containing the 3′-end of *lrgJ* and the 5′-end of *lrgK* was amplified using primers Mut.orf39–41I up/Mut.orf39–41I rp, digested with *Eco*RI and *Hin*dIII, and subcloned into the same sites of pUO9090, upstream of the apramycin resistance cassette, generating pUO-orf39–41I. Also, a 2.06 kb DNA fragment containing the 3′-end of *lrgM1*, *lrgA*, *lrgD*, and the 5′-end of *lrgN* was amplified using oligonucleotides Mut.orf39–41D up/Mut.orf39–41D rp, digested with EcoRV and XbaI and subcloned into the same sites of pUO-orf39–41I, downstream of the apramycin resistance gene, generating pUO-orf39–41. Finally, the whole fragment (5.5 kb) was rescued with SpeI and subcloned into the XbaI site of pHZ1358. pHZ-HMG: A 1.91 kb DNA fragment containing the 3′-end of *lrgT2*, *lrgE*, and the 5′-end of *lrgM2* was amplified using oligoprimers orf30HMG apra I up/orf30HMG apra I rp, digested with *Bgl*II and *Bam*HI, and subcloned in the right orientation into the same sites of pUO9090, upstream of the apramycin resistance cassette, generating pUO-HMGI. Also, a 2.02 kb DNA fragment containing the 3′-end of *lrgM2*, *lrgF*, and the 5′-end of *lrgG* was amplified using primers orf30HMG apra D up/orf30HMG apra D rp, digested with EcoRV and XbaI, and subcloned into the same sites of pUO-HMGI downstream of the apramycin resistance gene, generating pUO-HMG. Finally, the whole 5.5 kb DNA fragment was rescued with SpeI and subcloned into the XbaI site of pHZ1358. pHZ-LrgF: First, a 2.02 kb DNA fragment containing the 5′-end of *lrgE* and *lrgM2* and the 5′-end of *lrgF* was amplified using oligonucleotides orf31LnmF I2 up/orf31LnmF I2 rp, digested with *Hin*dIII and *Pst*I, and subcloned into the same sites of pUO9090, upstream of the apramycin resistance cassette, generating pUO-LnmFI. Then, a 2.04 kb DNA fragment containing the 3′-end of *lrgF* and the 5′-end of *lrgG* was amplified using primers orf31LnmF D2 up/orf31LnmF D2 rp, digested with EcoRV and XbaI, and subcloned into the same sites of pUO-LnmFI, downstream of the apramycin resistance cassette. Finally, the whole insert was released as a 5.5 kb SpeI fragment from the resultant construct pUO-LnmF and subcloned into the XbaI site of pHZ1358. pHZ-HalSyrB2: a 1.99 kb DNA fragment containing the 3′-end of *lrgP2*, *lrgS*, and the 5′-end of *lrgH* was amplified using oligonucleotides Hal.SyrB2.2 I up/Hal.SyrB2.2 I rp, digested with XbaI and *Bam*HI, and subcloned into the same sites of pUK21, generating pUK-HalSyrB2I. In addition, a 1.96 kb DNA fragment was amplified using Hal.SyrB2.2 D up/Hal.SyrB2.2 D rp as primers, digested with *Hin*dIII and *Bgl*II, and subcloned into the same sites of pUK-HalSyrB2I to generate pUK-HalSyrB2. Then, a hygromycin resistance gene was obtained as a *Pst*I-*Hin*dIII fragment from pLHyg and subcloned into the same sites of pUK-HalSyrB2. Finally, the 5.6 kb SpeI fragment was rescued and subcloned into the XbaI site of pHZ1358.

### Plasmid Constructs to Complement Mutant Strains

Genes were PCR amplified using specific primers (Table S1) and cloned under the control of the erythromycin resistance promoter ermEp*, in either plasmid pSETEH or pSETec. After introducing each plasmid in the corresponding mutant, the recombinant strains were then transformed with pEM4T-R2 to express the cluster specific activator *lrgR2* that is required to activate expression of the *lrg* BGC. The following plasmids were constructed as follows (Table 1). pSETEH-orf39–41: A 2.53 kb DNA fragment containing *lrgK*, *lrgL*, and *lrgM1* was amplified using oligonucleotides ermE39–41 up/ermE39–41 rp, digested with NheI and SpeI and subcloned in the right orientation into the XbaI site of pSETEH. pSETEH-HMG: A 1.35 kb DNA fragment containing *lrgM2* was amplified using oligonucleotides ermEorf30HMG up/ermEorf30HMG rp, digested with NheI and SpeI, and subcloned in the right orientation into the XbaI of pSETEH. pSETEH-LrgE-HMG: A 2.19 kb DNA fragment containing *lrgE* and *lrgM2* was amplified using ermEorf29LnmE up/ermEorf30HMG rp, digested with NheI and SpeI, and subcloned in the right orientation into the XbaI site of pSETEH. pSETEH-LrgF: An 871 bp DNA fragment containing *lrgF* was amplified using oligonucleotides ermEorf31LnmF up/ermEorf31LnmF rp, digested with NheI and SpeI, and subcloned in the right orientation into the XbaI site of pSETEH. pSETec-H: A 1.01 kb DNA fragment containing *lrgH* was amplified using primers ermEHalSyrB2 up/ermEHalSyrB2 rp, digested with NheI and SpeI, and subcloned in the right orientation, into the XbaI site of pSETec.

### UPLC Analysis and Purification of Largimycins

Extraction and UPLC analyses of LRGs were carried out as previously described.^11^ For purification purposes, strains were grown by a two-step culture method.^34^ In the production step, six 2-L Erlenmeyer flasks, each containing SM30a medium (400 mL), were incubated for 3 days for ΔlrgM2, 4 days for ΔlrgKLM1, and 5 days for ΔlrgH. To purify compounds, cultures were extracted with ethyl acetate plus formic acid, the organic extracts dried down under a vacuum, and the residues dissolved in a small volume of DMSO/methanol (1:1). Products were purified by semipreparative HPLC using a SunFireC18 column (10 mm, 10 × 150 mm, Waters). Compounds were chromatographed with mixtures of acetonitrile or methanol and 0.05% TFA in water, under isocratic conditions optimized for each compound, at 5 mL/min. The purification procedure afforded LRG K1 (2.7 mg), LRG K2 (0.6 mg), LRG K3 (0.2 mg), LRG K4 (0.9 mg), LRG M1 (1.3 mg), LRG M2 (5.3 mg), LRG M3 (4.4 mg), LRG H1 (0.6 mg), and LRG H2 (0.8 mg).

### Spectroscopic Analysis and Structural Elucidation of Largimycins

Structural elucidation of each compound was carried out by ESI-TOF mass spectrometry and NMR spectroscopy, further assisted by comparisons with the reported spectroscopic data of known LRGs.^11^ HRMS spectra were collected by LC-MS analyses using an Agilent 1200RR HPLC equipped with a SB-C8 column (2.1 × 30 mm, Zorbax) and coupled to a Bruker maXis Spectrometer. Chromatographic and ionization conditions were identical to those previously described.^35,36^ UV/vis (DAD) spectra were also collected in the same chromatographic analyses. NMR spectra were recorded in DMSO-*d*_6_ at 24 °C on a Bruker AVANCE III-500 MHz (500 and 125 MHz for ^1^H and ^13^C NMR, respectively) equipped with a 1.7 mm TCI MicroCryoProbe, using the residual solvent signal as an internal reference (δ_H_ 2.50 and δ_C_ 39.5). Key correlations observed in the COSY and HMBC spectra combined with the determined molecular formulas rendered the full connectivity of the compounds. For LRG H1 and LRG H2, molecular modeling was used in combination with NMR data to determine the relative configurations, using 3D structural models generated with Chem3D Pro 12.0 starting from the molecular models of LRG A1, LRG A2, and LRG A4,^11^ which were based on the reported X-ray structure of LNM E2.^37^ The structures were first constructed to roughly satisfy the observed ^3^*J*_HH_ and key NOESY correlations and then submitted to energy-minimization by molecular mechanics with the MM2 force field using as gradient convergence criteria an RMS value of 0.001. Molecular modeling images (see Supporting Information) were generated with PyMOL.^38^

## Supporting Information Available

Generation and characterization of mutant strains; LC-HRMS analysis of compounds in culture supernatants; spectroscopic data and structural elucidation of LRG K1–K4, LRG M1–M3, and LRG H1–H2; oligonucleotides used for PCR amplifications (PDF)
