# Supplementary material for: Biosynthesis of Largimycins in Streptomyces argillaceus Involves Transient β-Alkylation and Cryptic Halogenation Steps Unprecedented in the Leinamycin Family
Source: ACS Chem Biol. 2022 Jul 13;17(8):2320–31. doi: 10.1021/acschembio.2c00416 (PMC9396626; doi:10.1021/acschembio.2c00416)
Supplement: Supplementary file 1 — cb2c00416_si_001.pdf [file cb2c00416_si_001.pdf]

SUPPORTING INFORMATION

Biosynthesis of largimycins in *Streptomyces argillaceus* involves transient β-alkylation and cryptic halogenation steps unprecedented in the leinamycin family

Adriana Becerril,[†,‡] Ignacio Pérez-Victoria,[#] Jesús Martín,[#] Fernando Reyes,[#] Jose A. Salas[†,‡] and Carmen Méndez*[,†,‡]

[†]Departamento de Biología Funcional e Instituto Universitario de Oncología del Principado de Asturias (I.U.O.P.A), Universidad de Oviedo, 33006 Oviedo, Spain

[‡]Instituto de Investigación Sanitaria de Asturias (ISPA), 33011 Oviedo, Spain

[#]Fundación MEDINA, Centro de Excelencia en Investigación de Medicamentos Innovadores en Andalucía, Armilla, 18016 Granada, Spain.

*** Correspondence:** Carmen Méndez (cmendezf@uniovi.es)

Structure elucidation of largimycins

Largimycin K1 (LRG K1, **1**) was assigned a molecular formula $C_{23}H_{31}ClN_2O_6$ based on the observed ion $[M+H]^+$ at *m/z* 467.1952 (calcd. for $C_{23}H_{32}ClN_2O_6^+$ = 467.1943, □m = 1.9 ppm) alongside their corresponding monochlorinated isotopic pattern, indicating 9 degrees of unsaturation. The structure of **1** was determined by detailed 1D (^{1}H) and 2D NMR (COSY, NOESY, HSQC and HMBC) spectroscopic analyses further assisted by comparison with the NMR data of already reported LRGs.[11] Interpretation of the HSQC and HMBC spectra revealed the presence of 6 quaternary carbons (including two ester/amide carbonyls in the range δ_C 169-174, and four sp^2 carbons at δ_C 136.6, 138.3, 145.0 and 162.1), 9 methines (including six olefinic/aromatic carbons, two oxygenated methines and one methine likely corresponding to the CH_α of an amino acid moiety), one olefinic methylene, 4 aliphatic methylenes and one methyl group (corresponding to an *N*-acetyl group). Analysis of COSY correlations identified different spin systems which could be connected using the key long-range correlations observed in the HMBC spectrum (Figure S14). To meet the determined molecular formula, the chlorine atom was easily assigned as a C19 substituent based on the observed chemical shifts for that methylene, rendering a formal γ-chlorinated threonine residue. The spin system comprising H10 to H13, contains four olefinic protons corresponding to Δ^{10} *E* and Δ^{12} *Z* double bonds, as indicated by the measured coupling constants of 15.5 and 11.4 Hz, respectively. The Δ^{10} *E* double bond of this spin system is conjugated with an olefinic methylene group, as indicated by the key long-range correlations between H11/C9 and H10/C20. On the Δ^{12} *Z* double bond end, this spin system is conjugated with the aromatic oxazole heterocycle characteristic of LRGs, as revealed by the key HMBC correlations between H15 and C13, C14 and C16. The extended π-system between positions 9 and 16 is connected to another spin system, comprising H7 and H8, as demonstrated by the long-range correlations from H10 to C8 and from H8 to C9. The HMBC correlations of the methyl H21 with C5, C6 and C7 confirmed the expected substitution position of this methyl group. As expected, the chemical shifts from position 5 to 17 were indeed very similar to those of LRG O1,[11] which shares identical connectivity in that segment. The terminal spin system between H5 and H2 was connected to a free carboxylic acid functional group at C1 via the HMBC correlation from H2 to the C1 carbonyl. Contrary to LRG O1, this carboxylate does not participate in a macrocyclic ring closure through amide bond with the formal amino group at C17, which is N-acetylated as demonstrated by the key HMBC correlations between the amido hydrogen (17-NH) and the acetyl methyl and carbonyl carbons 1' and 2'. The *E* stereochemistry of the Δ^6 double bond was assigned based on comparisons with LRG O1,[11] and further corroborated by the strong NOE observed between H21 and H8 (Figure S14). The absolute configuration at C17 and C18 has been assigned to be the same as those found in L-Thr, LNMs and

LRGs, based on biosynthetic and phylogenetic arguments,[12] as already described for the first LRGs discovered.[11] The absolute configuration at the hydroxylated position 3 was not determined.

Largimycin K2 (LRG K2, **2**) was assigned the molecular formula $C_{21}H_{27}ClN_2O_5$ based on the observed ion $[M+H]^+$ at *m/z* 423.1686 (calcd. for $C_{21}H_{28}ClN_2O_5^+$ = 423.1681, Δm = 1.2 ppm) alongside their corresponding isotopic pattern, indicating 9 degrees of unsaturation. The structure of **2** was determined by detailed 1D (^{1}H) and 2D NMR (COSY, NOESY, HSQC and HMBC) spectroscopic analyses further assisted by comparison with the NMR data of LRG K1 (**1**). Interpretation of the spectra revealed identical NMR features as those observed in **1** but the lack of one aliphatic methylene and one hydroxylated methine group, perfectly accounting for the formal "C_2H_4O" difference between both molecular formulas. Key COSY and long-range HMBC correlations (Figure S14) rendered a structure almost identical to **1** lacking the two terminal carbons, C1 and C2 and turning C3 into a free carboxylic acid group. Stereochemistry of the double bonds was determined as described above for **1**.

Largimycin K3 (LRG K3, **3**) was assigned the molecular formula $C_{22}H_{31}ClN_2O_4$ based on the observed ion $[M+H]^+$ at *m/z* 423.2047 (calcd. for $C_{22}H_{32}ClN_2O_4^+$ = 423.2045, Δm = 0.5 ppm) alongside their corresponding isotopic pattern, indicating 8 degrees of unsaturation. The structure of **2** was determined by detailed 1D (^{1}H) and 2D NMR (COSY, HSQC and HMBC) spectroscopic analyses further assisted by comparison with the NMR data of LRG K1 (**1**). Although the NMR sample contained significant impurities, careful interpretation of the spectra revealed identical NMR features as those observed in **1** but the lack of the free carboxylic acid group and one aliphatic methylene concomitant with the presence of a new methyl as first carbon atom of the chain. Such differences perfectly accounted for the formal "CO_2" difference between both molecular formulae. Key COSY and long-range HMBC correlations (Figure S14) rendered a structure corresponding formally to a decarboxylated form of **1**. Stereochemistry of the double bonds was assigned based on the measured coupling constants and chemical shift comparisons with **1** since the NOESY spectrum was not acquired. The absolute configuration at the hydroxylated position 3 was not determined but must be identical to that in **1** based on their common biosynthetic origin.

Largimycin K4 (LRG K4, **4**) was assigned the molecular formula $C_{22}H_{29}ClN_2O_4$ based on the observed ion $[M+H]^+$ at *m/z* 421.1888 (calcd. for $C_{22}H_{30}ClN_2O_4^+$ = 421.1889, Δm = 0.2 ppm) alongside their corresponding isotopic pattern, indicating 9 degrees of unsaturation. The structure of **2** was determined by detailed 1D (^{1}H) and 2D NMR (COSY, NOESY, HSQC and HMBC) spectroscopic analyses further assisted by comparison with the NMR data of LRG K1 (**1**) and LRG K3 (**3**). Interpretation of the spectra revealed identical NMR features as those observed in **3** but the lack of one hydroxylated methine group concomitant with the presence of a new ketone group. Such differences perfectly accounted for the formal "H_2" difference between the molecular formulas of **4** and

3, suggesting **4** as an identical compound to **3** but with a higher oxidation state at C3 (ketone vs. alcohol). Key COSY and long-range HMBC correlations (Figure S14) confirmed the expected connectivity. Stereochemistry of the double bonds was determined as described above for **1**.

Largimycin M1 (LRG M1, **5**) was assigned the molecular formula $C_{16}H_{17}ClN_2O_5$ based on the observed ion $[M+H]^+$ at *m/z* 353.0896 (calcd. for $C_{16}H_{18}ClN_2O_5^+$ = 353.0899 Δm = 0.8 ppm) alongside their corresponding monochlorinated isotopic pattern, indicating 9 degrees of unsaturation. The structure of **5** was determined by detailed 1D (^1H) and 2D NMR (COSY, NOESY, HSQC and HMBC) spectroscopic analyses further assisted by comparison with the NMR data of already reported LRGs[11] and LRG K1 (**1**). Interpretation of the HSQC and HMBC spectra revealed the presence of 6 quaternary carbons (including one ester/amide carbonyl at δ_C 164.4 and five sp^2 carbons in the range δ_C 99-164), 8 methines (including six olefinic/aromatic carbons, one oxygenated methine and one methine likely corresponding to the CH_α of an amino acid moiety), one olefinic methylene, one aliphatic methylene and a methyl group (as an olefinic double bond substituent). Analysis of COSY correlations identified different spin systems which could be connected using the key long-range correlations observed in the HMBC spectrum (Figure S15). To meet the determined molecular formula, the chlorine atom was again easily assigned as a C19 substituent based on the observed chemical shifts for that methylene, rendering a formal γ-chlorinated threonine residue. The spin system comprising H10 to H13, contains four olefinic protons corresponding to two *E* double bonds, as indicated by the measured coupling constants and key NOEs (Figure S15). This spin system is conjugated on the H13 end with the aromatic oxazole heterocycle characteristic of LRGs, as revealed by the key HMBC correlations between H15 and C13, C14 and C16. On the H10 end, the mentioned spin system is conjugated to an □-pyrone moiety, as revealed by the key HMBC correlation between H13 and C8, C9, between H8 and C9,C7, C6, and between H21 and C5, C6, C7. Such α-pyrone motif accounts for the difference observed in the UV spectrum of **5** compared to that of known LRGs an the previously described LRGs K1, K2, K3 and K4. Once again, the absolute configuration at C17 and C18 has been assigned to be the same as those found in L-Thr, based on the same arguments as indicated for the previous LRGs.

Largimycin M2 (LRG M2, **6**) was assigned the molecular formula $C_{16}H_{17}ClN_2O_5$ based on the observed ion $[M+H]^+$ at *m/z* 353.0897 (calcd. for $C_{16}H_{18}ClN_2O_5^+$ = 353.0899 Δm = 0.6 ppm) alongside their corresponding isotopic pattern, indicating 9 degrees of unsaturation. The molecular formula is identical to that of LRG M1 (**5**). The structure of **6** was determined by detailed 1D (^1H) and 2D NMR (COSY, NOESY, HSQC and HMBC) spectroscopic analyses further assisted by comparison with the NMR data of LRG M1 (**5**) and LRG K1 (**1**). Interpretation of the spectra revealed identical NMR features as those observed in **5** but with important differences in the chemical shifts of the olefinic protons. Key COSY and long-range HMBC correlations (Figure S15) confirmed identical connectivity to that LRG

M1 (**5**) but differing in the stereochemistry of the Δ^{12} double bond, being now *Z* (as opposed to the E stereochemistry found in **5**), as revealed by the coupling constant of 11.1 Hz between H12 and H13, thus following the trend of all LRG described before but LRG M1.

Largimycin M3 (LRG M3, **7**) was assigned the molecular formula $C_{18}H_{19}ClN_2O_6$ based on the observed ion $[M+H]^+$ at *m/z* 395.1002 (calcd. for $C_{18}H_{20}ClN_2O_6^+$ = 395.1004 Δm = 0.5 ppm) alongside their corresponding isotopic pattern, indicating 10 degrees of unsaturation. The structure of **7** was determined by detailed 1D (^{1}H) and 2D NMR (COSY, NOESY, HSQC and HMBC) spectroscopic analyses further assisted by comparison with the NMR data of LRG M1 (**5**). Interpretation of the spectra revealed identical NMR features as those observed in **5** with the additional presence of an *N*-acetyl group (in the amino group of the Thr residue) which perfectly accounts for the formal "C_2H_2O" difference between the molecular formulae of **7** and **5**. Key COSY and long-range HMBC correlations (Figure S15) confirmed the structure of LRG M3 (**5**) as an *N*-acetylated version of LRG M1 (**5**). Stereochemistry of the double bonds was determined as described above for **5**.

Largimycin H1 (LRG H1, **8**) was assigned the molecular formula $C_{23}H_{26}N_2O_9S$ based on the observed ion $[M+NH_4]^+$ at *m/z* 524.1700 (calcd. for $C_{23}H_{30}N_3O_9S^+$ = 524.1697, Δm = 0.6 ppm) alongside its corresponding isotopic pattern, indicating 12 degrees of unsaturation. The structure of **8** was established after detailed 1D (^{1}H) and 2D NMR (COSY, NOESY, HSQC and HMBC) spectroscopic analyses further assisted by comparisons with the NMR data of LRG A4 and LRG A1.[11] The NMR spectroscopic data of **8** (Table S9 and Figure S12) were remarkably similar to those of LRG A4 with an important difference, the epoxide resonances corresponding to positions 18 and 19 in LRG A4 are absent in **8** which rather displays one methyl and one hydroxylated methine signals at those positions (corresponding to a formal Thr residue). The pattern of COSY and HMBC correlations of **8** (Figure S16) corroborated the almost identical connectivity of LRG H1 and LRG A4**.** The oxime double bond was assigned a *Z* stereochemistry based the observed δ_C 68.2 for C18 and comparison with the empirical chemical shift prediction obtained for the two possible *E*/*Z* configurations of the oxime double bond, as it was described for the first LRGs discovered.[11] Not surprisingly, the NOESY correlations observed for **8** (Figure S16) also match those found in LRG A1 and A4 **1**, indicating the expected identical relative configuration. The absolute configuration of the chiral centers at C3 and C18 was assigned to be the same as for the previously reported LRGs, based on their common biosynthetic origin and the phylogenetic arguments already described for the first LRGs discovered.[11]

Largimycin H2 (LRG H2, **9**) was assigned the molecular formula $C_{28}H_{33}N_3O_{11}S_2$ based on the observed ion $[M+NH_4]^+$ at *m/z* 669.1894 (calcd. for $C_{28}H_{34}N_3O_{11}S_2^+$ = 669.1895, Δm = 0.1 ppm) alongside its corresponding isotopic pattern, indicating 14 degrees of unsaturation. The extra sulfur atom in the molecular formula, compared to **8** suggested the presence of a CysNAc moiety as found

in LRG A1 and LRG A2. The structure of **9** was established after detailed 1D (^{1}H) and 2D NMR (COSY, NOESY, HSQC and HMBC) spectroscopic analyses further assisted by comparisons with the NMR data of LRG A2[11] and LRG H1. The NMR spectroscopic data of **9** (Table S10 and Figure S13) were remarkably similar to those of LRG A2 with again the same difference as between LRG H1 and LRG A4: the epoxide resonances corresponding to positions 18 and 19 in LRG A2 are absent in **9** which rather displays one methyl and one hydroxylated methine signals at those positions (corresponding to a formal Thr residue), as found in LRG H1. The pattern of COSY and HMBC correlations of **8** (Figure S16) corroborated the almost identical connectivity of LRG H2 and LRG A2**.** The oxime double bond was assigned a *Z* stereochemistry based the observed δ_C 67.7 for C18 and comparison with the empirical chemical shift prediction obtained for the two possible *E*/*Z* configurations of the oxime double bond, as indicated before for LRG H1 and it was described for the first LRGs discovered.[11] Not surprisingly, the NOESY correlations observed for **9** (Figure S15) also match those found in LRG A2, indicating the expected identical relative configuration. The absolute configuration of the chiral centers at C3 and C18 was assigned to be the same as for LRG H1, based on their common biosynthetic origin, and the absolute configuration of position 2’ of the S-conjugated CysNAc unit was assigned to be *S*, since this moiety derives from mycothiol, as described for the first LRGs discovered.[11]

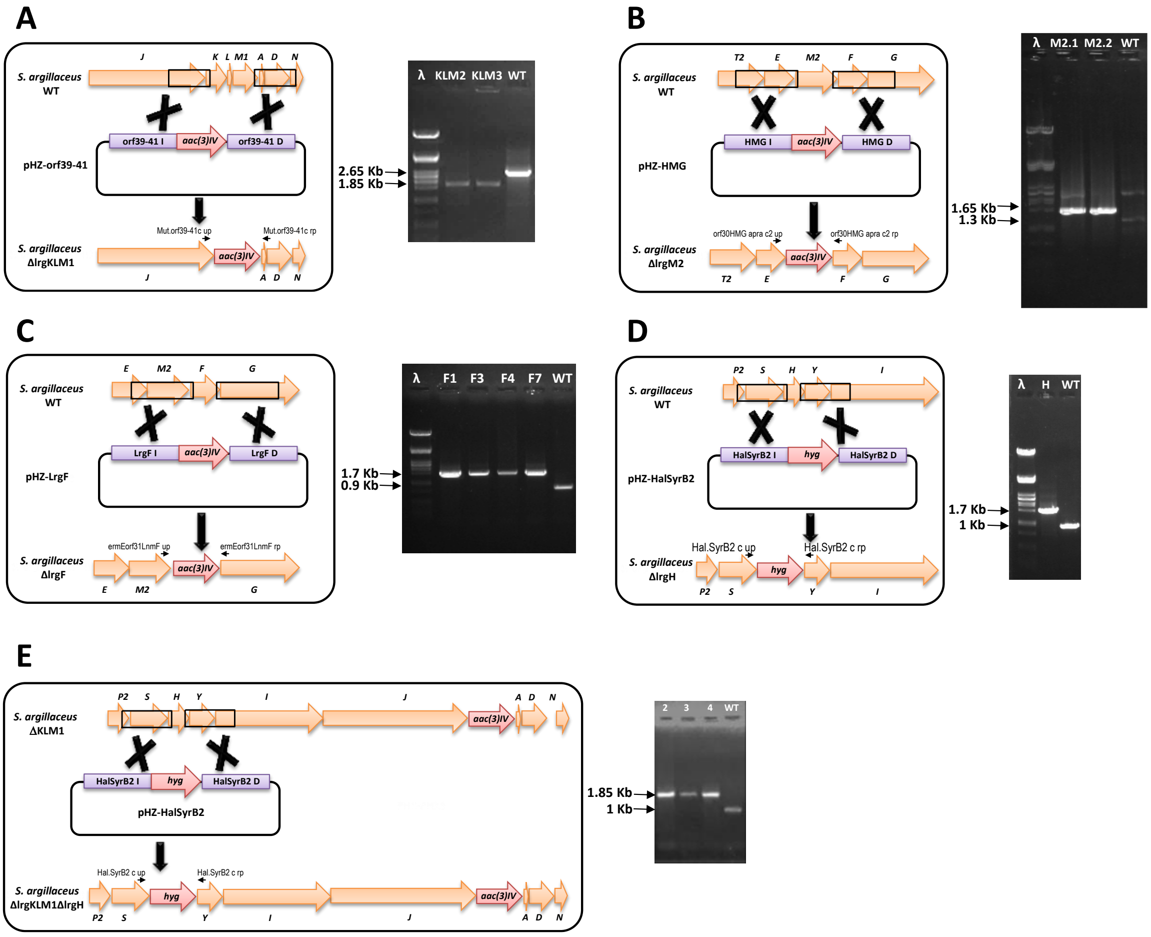

Figure S1. Confirmation of *Streptomyces argillaceus* mutants. Generation of *Streptomyces argillaceus* mutants in *lrg*. Each panel includes a scheme representing construction of a mutant and its confirmation by PCR. (A) *S. argillaceus* ΔlrgKLM1; (B) *S. argillaceus* ΔlrgM2; (C) *S. argillaceus* ΔlrgF; (D) *S. argillaceus* ΔlrgH; (E) *S. argillaceus* ΔlrgKLM1ΔlrgH. WT, wild type strain; *aac(3)IV*, apramycin resistance gene. λ, Pst-digested Lambda DNA.

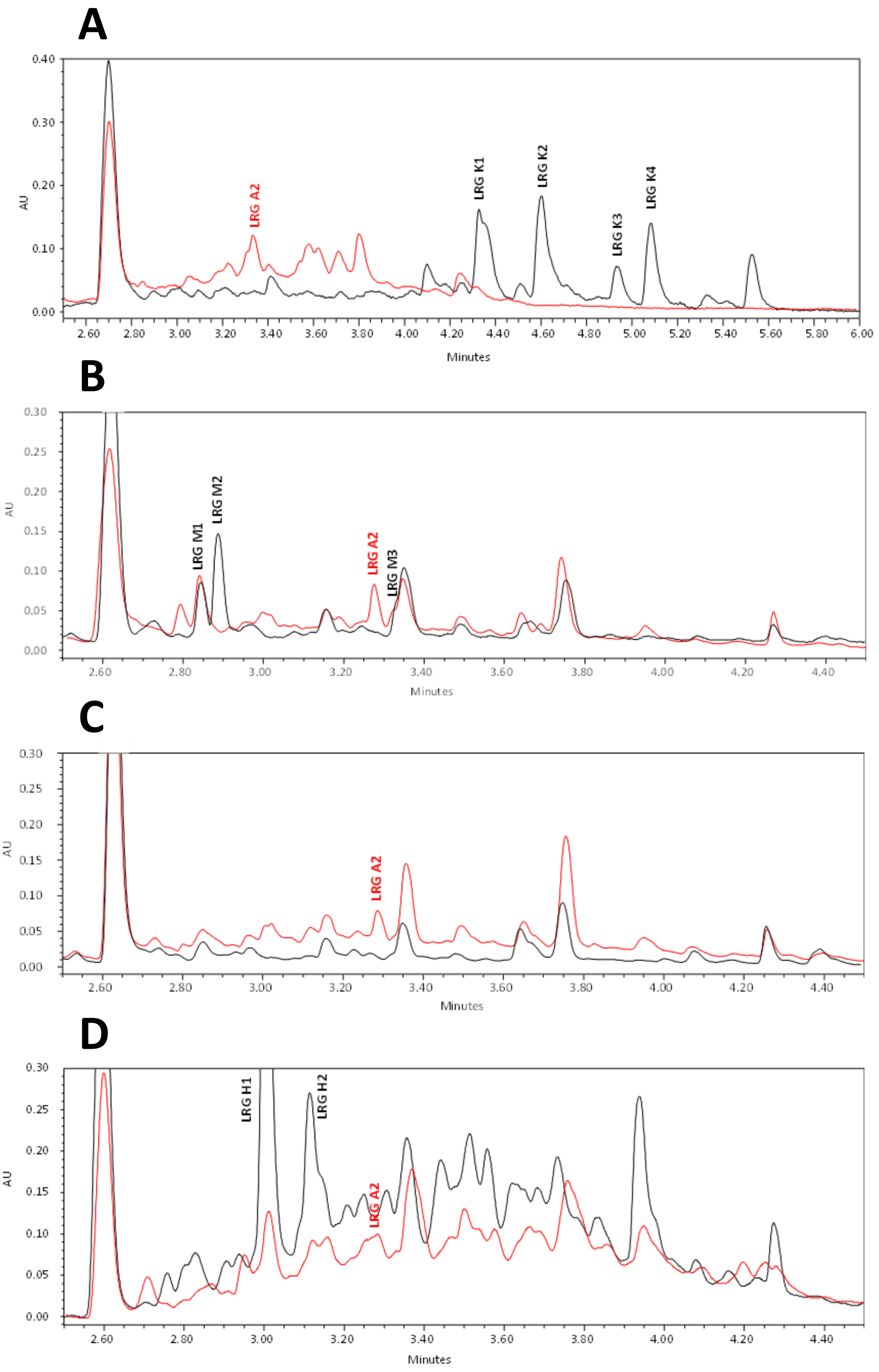

Figure S2: UPLC analyses of extracts of complemented *S. argillaceus lrg* mutants. Chromatograms at 330 nm of extracts from complemented mutants (in red) in comparison to the corresponding mutant (in black): (A) *S. argillaceus* ΔlrgKLM1-R2-KLM1; *S. argillaceus* ΔlrgM2-R2-EM2; (C) *S. argillaceus* ΔlrgF-R2-F; (D) *S. argillaceus* ΔlrgH-R2-H. Peak corresponding to LRG A2 is indicated, as well as peaks produced by the mutant strains.

A

B

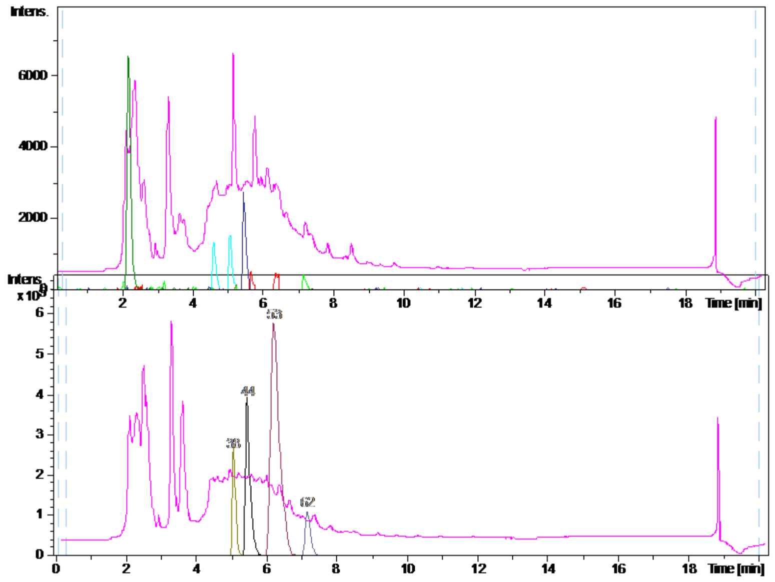

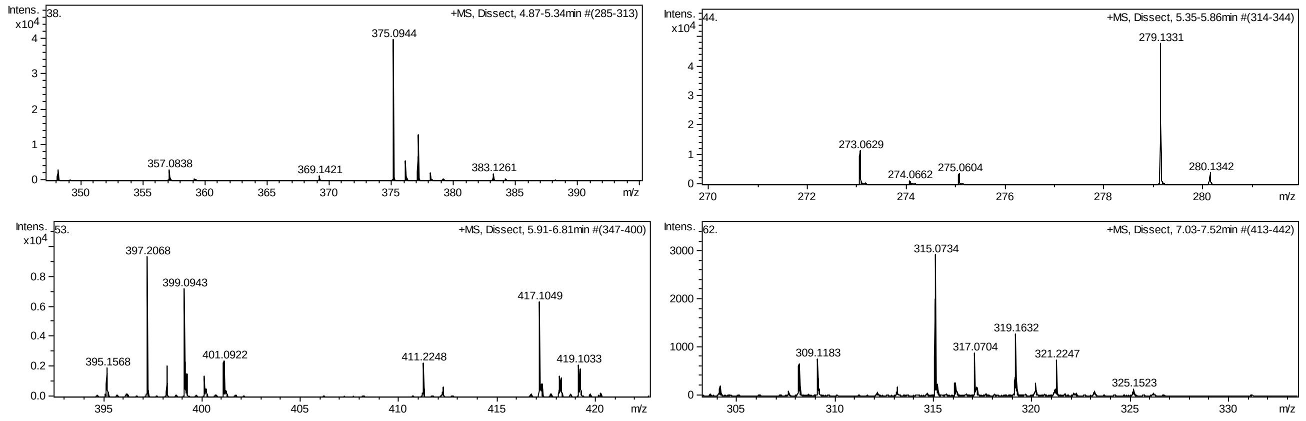

Cmpd 38: $C_{15}H_{19}ClN_2O_7$ based on the observed ion $[M+H]^+$ at *m/z* 375.0944 (calcd. for $C_{15}H_{20}ClN_2O_7{}^+$ = 375.0954, Δm = 3 ppm)

Cmpd 44: $C_{11}H_{13}ClN_2O_4$ based on the observed ion $[M+H]^+$ at *m/z* 273.0629 (calcd. for $C_{11}H_{14}ClN_2O_4{}^+$ = 273.0637, Δm = 3 ppm)

Cmpd 53: $C_{17}H_{21}ClN_2O_8$ based on the observed ion $[M+H]^+$ at *m/z* 417.1049 (calcd. for $C_{17}H_{21}ClN_2O_8{}^+$ = 417.1053, Δm = 1 ppm)

Cmpd 62: $C_{13}H_{15}ClN_2O_5$ based on the observed ion $[M+H]^+$ at *m/z* 315.0734 (calcd. for $C_{15}H_{20}ClN_2O_7{}^+$ = 315.0742, Δm = 3 ppm)

Figure S3: LC-HRMS analysis of chlorinated compounds identified in culture supernatants of *S. argillaceus* ΔlrgF-R2. Analysis of (A) *S. argillaceus* WT-R2 and (B) *S. argillaceus* ΔlrgF-R2

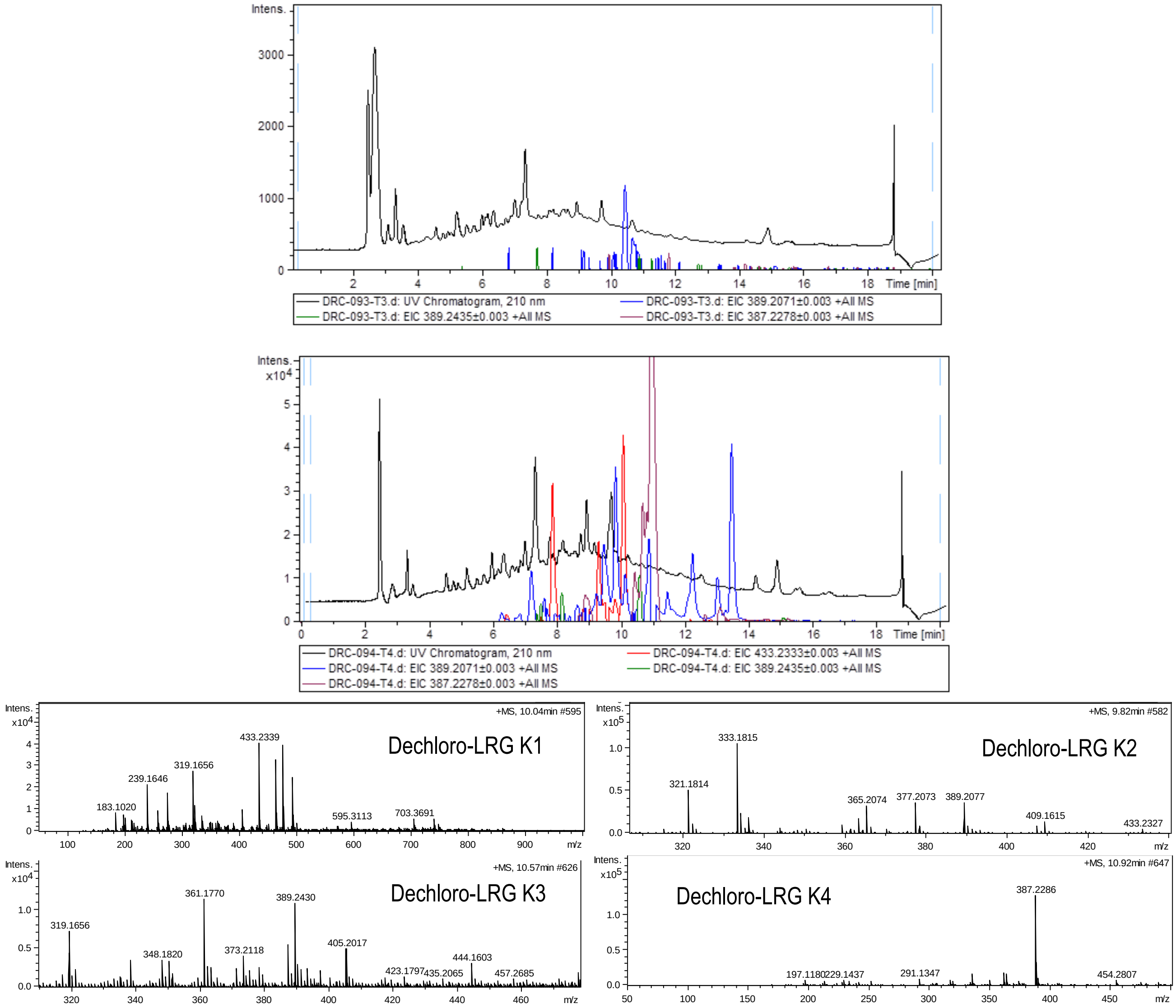

Dechloro-LRG K1: $C_{23}H_{32}N_2O_6$ based on the observed ion $[M+H]^+$ at *m/z* 433.2339 (calcd. for $C_{23}H_{33}N_2O_6^+$ = 433.2333, Δm = 1 ppm)

Dechloro-LRG K2: $C_{21}H_{28}N_2O_5$ based on the observed ion $[M+H]^+$ at *m/z* 389.2077 (calcd. for $C_{21}H_{29}N_2O_5^+$ = 389.2071, Δm = 2 ppm)

Dechloro-LRG K3: $C_{22}H_{32}N_2O_4$ based on the observed ion $[M+H]^+$ at *m/z* 389.2430 (calcd. for $C_{22}H_{32}N_2O_4^+$ = 389.2435, Δm = 1 ppm)

Dechloro-LRG K4: $C_{22}H_{30}N_2O_4$ based on the observed ion $[M+H]^+$ at *m/z* 387.2286 (calcd. for $C_{22}H_{31}N_2O_4^+$ = 387.2278, Δm = 2 ppm)

Figure S4. LC-HRMS analysis of dechlorinated compounds identified in culture supernatants of *S. argillaceus* ΔlrgKLM1-ΔlrgH-R2. Analysis of (A) *S. argillaceus* WT-R2 and (B) *S. argillaceus* ΔlrgKLM1-ΔlrgH-R2. Ion extraction was employed to detect dechlorinated LRG K1-K4.

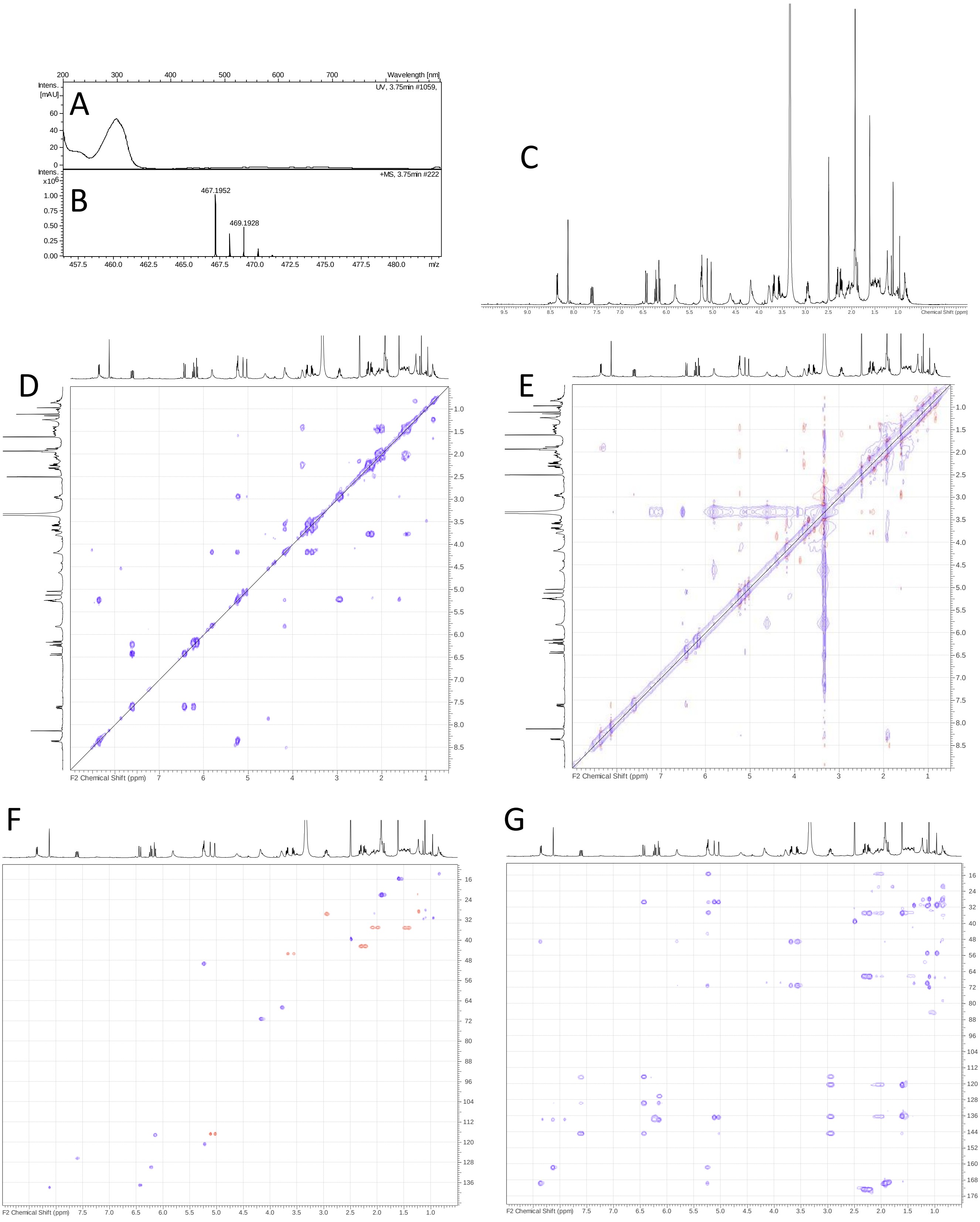

Figure S5. Spectroscopic data of LRG K1 (1): (A) UV-DAD spectrum; (B) HRMS spectrum; (C) 1H NMR spectrum in DMSO-d6, 500 MHz; (D) COSY spectrum; (E) NOESY spectrum; (F) Edited HSQC spectrum; (G) HMBC spectrum.

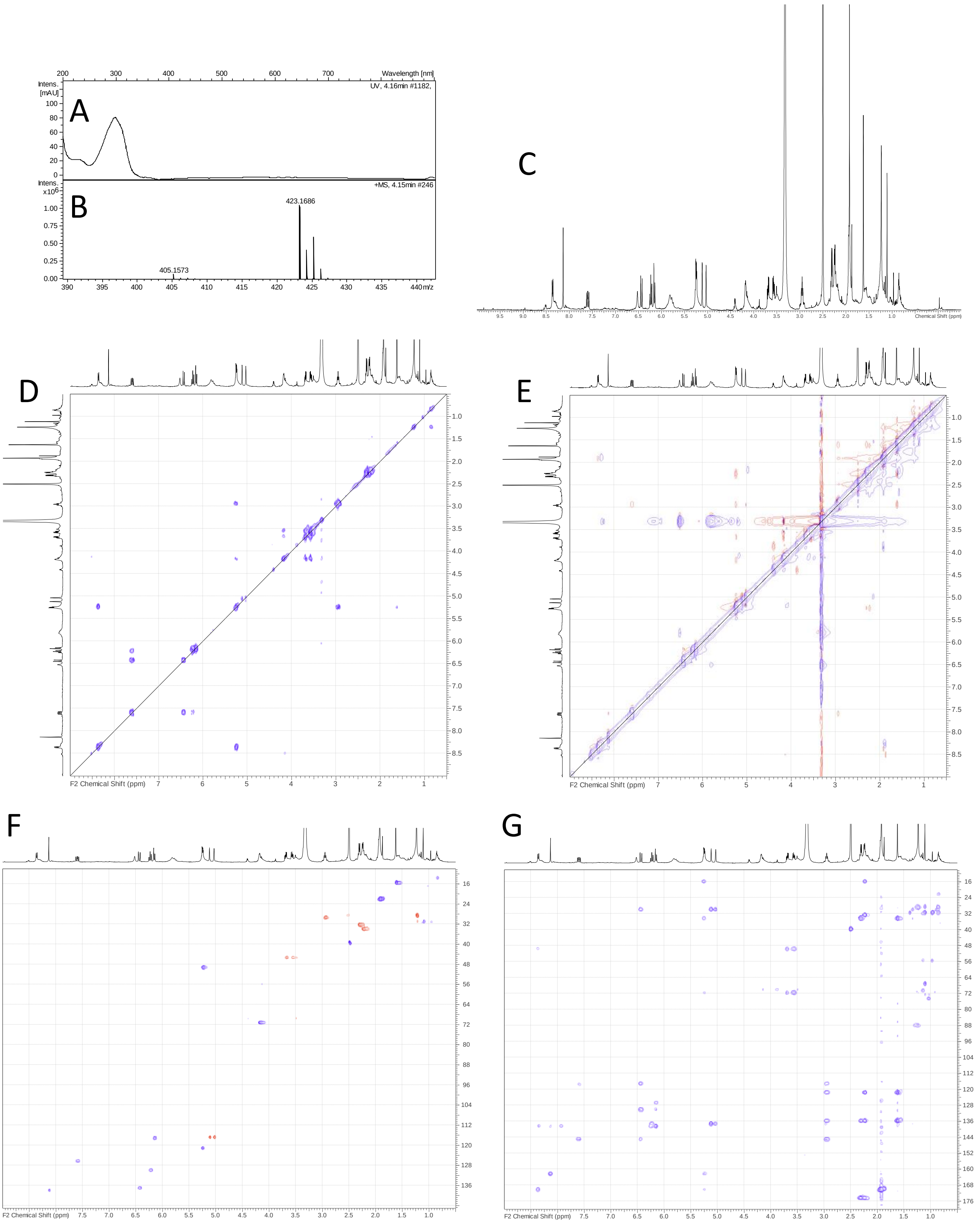

Figure S6. Spectroscopic data of LRG K2 (2): (A) UV-DAD spectrum; (B) HRMS spectrum; (C) 1H NMR spectrum in DMSO-d6, 500 MHz; (D) COSY spectrum; (E) NOESY spectrum; (F) Edited HSQC spectrum; (G) HMBC spectrum.

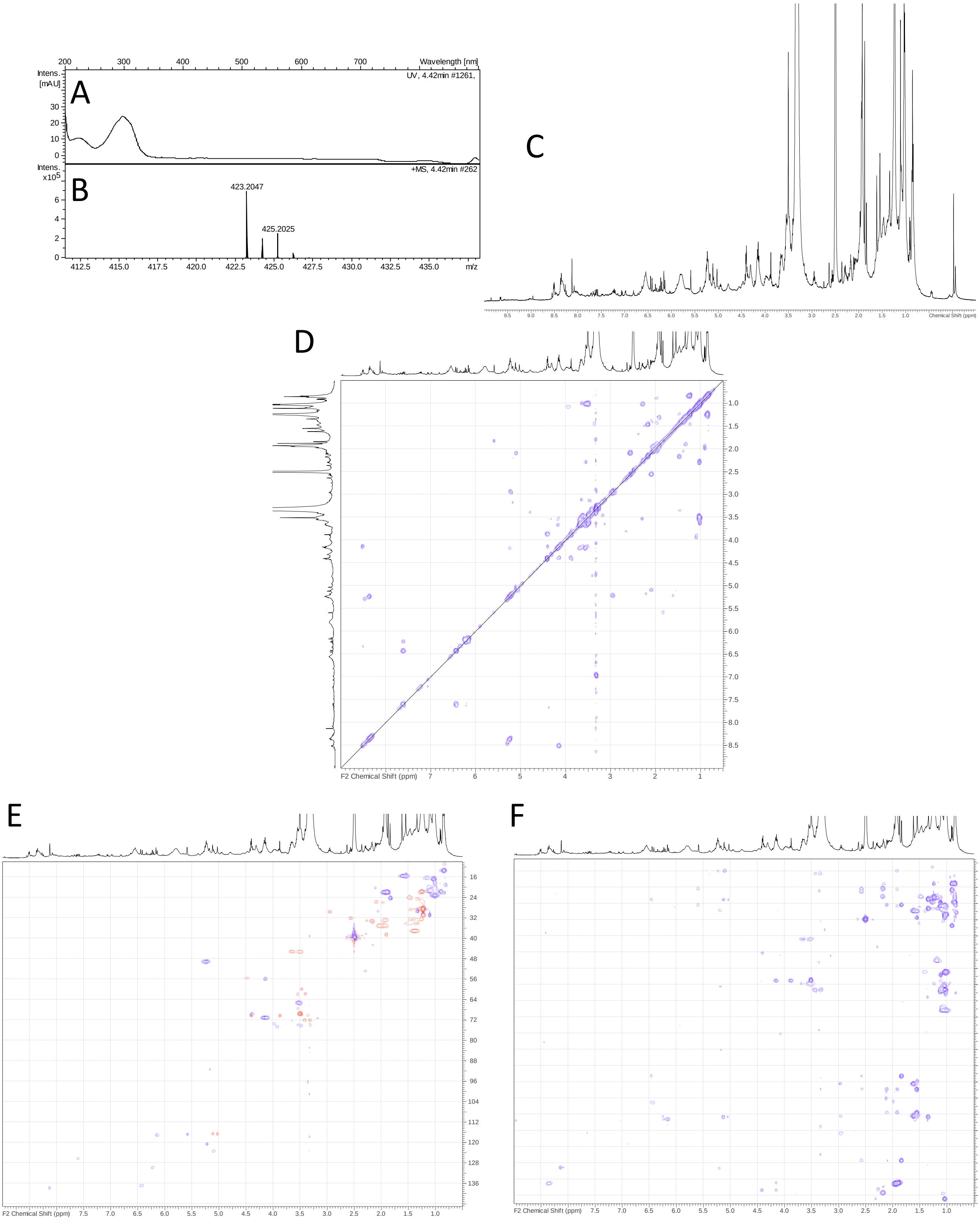

Figure S7. Spectroscopic data of LRG K3 (3): (A) UV-DAD spectrum; (B) HRMS spectrum; (C) 1H NMR spectrum in DMSO-d6, 500 MHz; (D) COSY spectrum; (E) Edited HSQC spectrum; (F) HMBC spectrum.

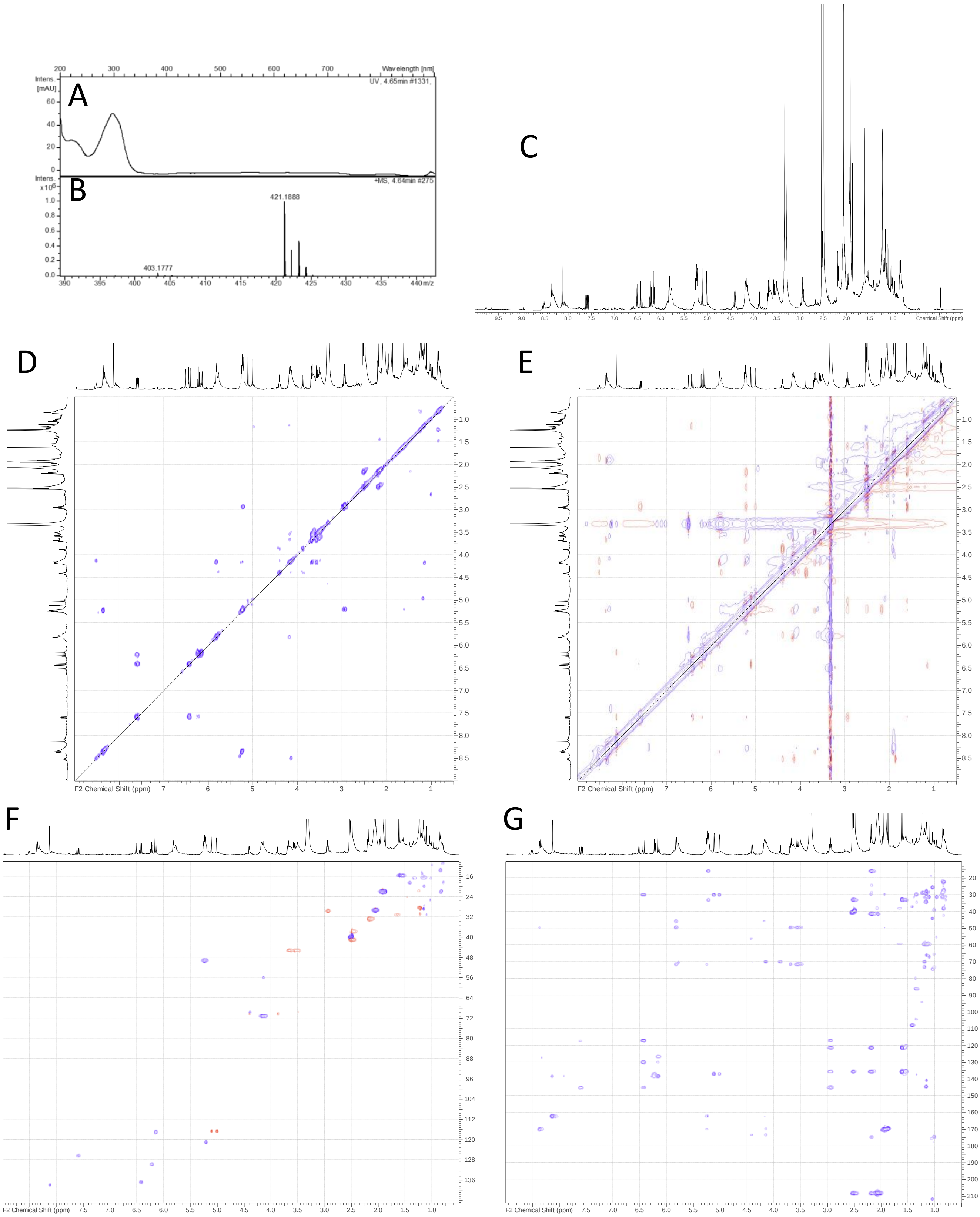

Figure S8. Spectroscopic data of LRG K4 (4): (A) UV-DAD spectrum; (B) HRMS spectrum; (C) 1H NMR spectrum in DMSO-d6, 500 MHz; (D) COSY spectrum; (E) NOESY spectrum; (F) Edited HSQC spectrum; (G) HMBC spectrum

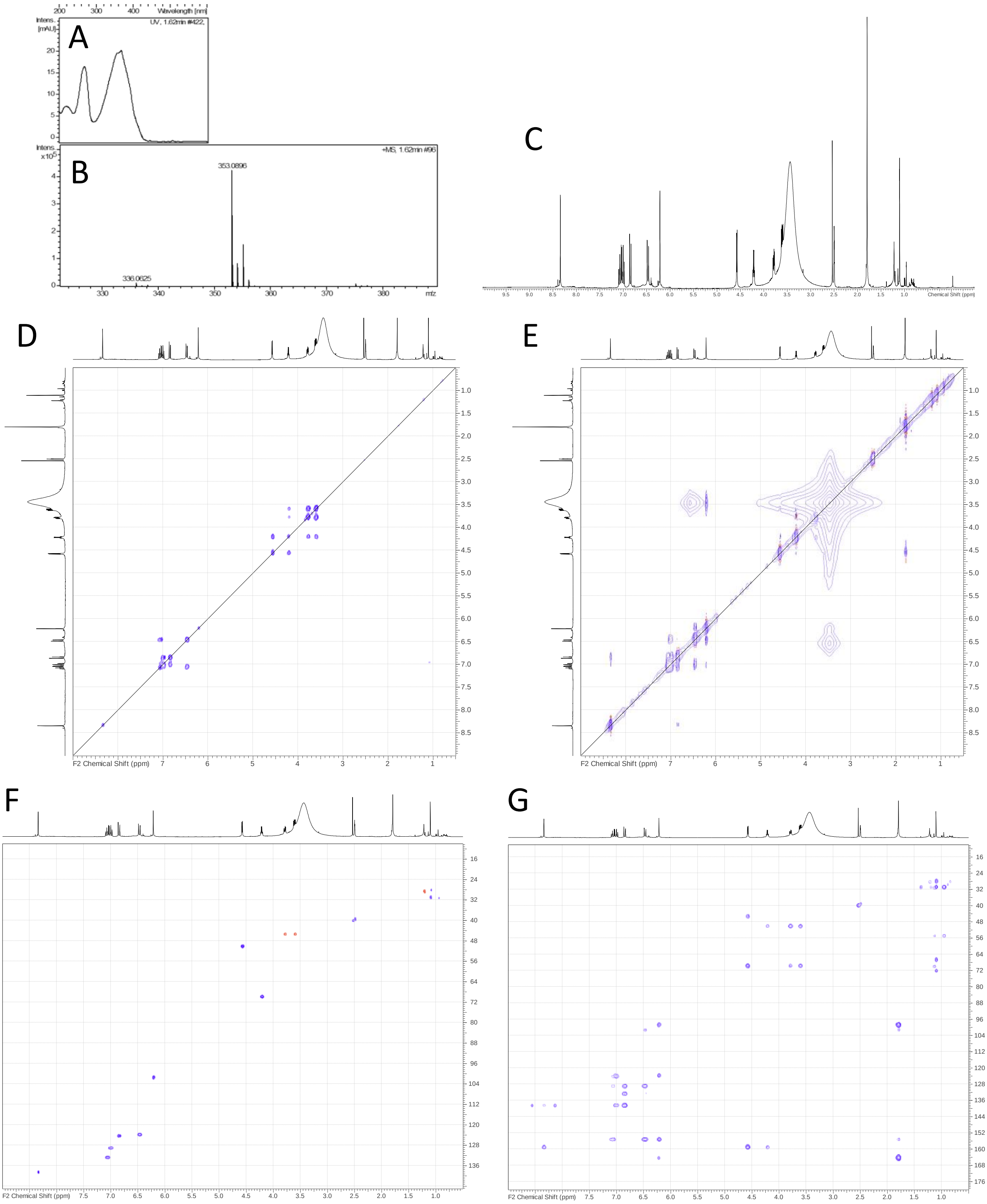

Figure S9. Spectroscopic data of LRG M1 (5): (A) UV-DAD spectrum; (B) HRMS spectrum; (C) 1H NMR spectrum in DMSO-d6, 500 MHz; (D) COSY spectrum; (E) NOESY spectrum; (F) Edited HSQC spectrum; (G) HMBC spectrum

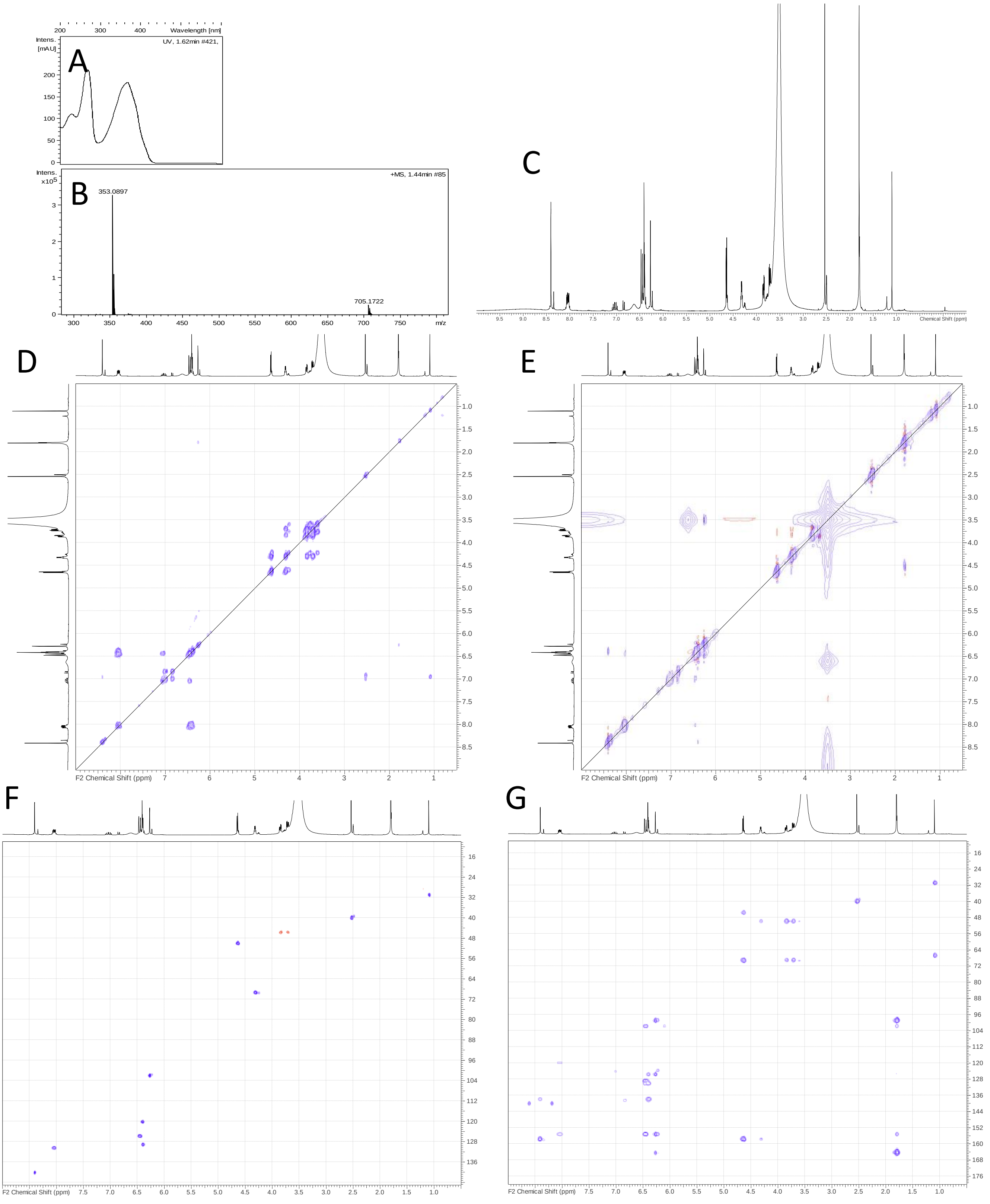

Figure S10. Spectroscopic data of LRG M2 (6): (A) UV-DAD spectrum; (B) HRMS spectrum; (C) 1H NMR spectrum in DMSO-d6, 500 MHz; (D) COSY spectrum; (E) NOESY spectrum; (F) Edited HSQC spectrum; (G) HMBC spectrum

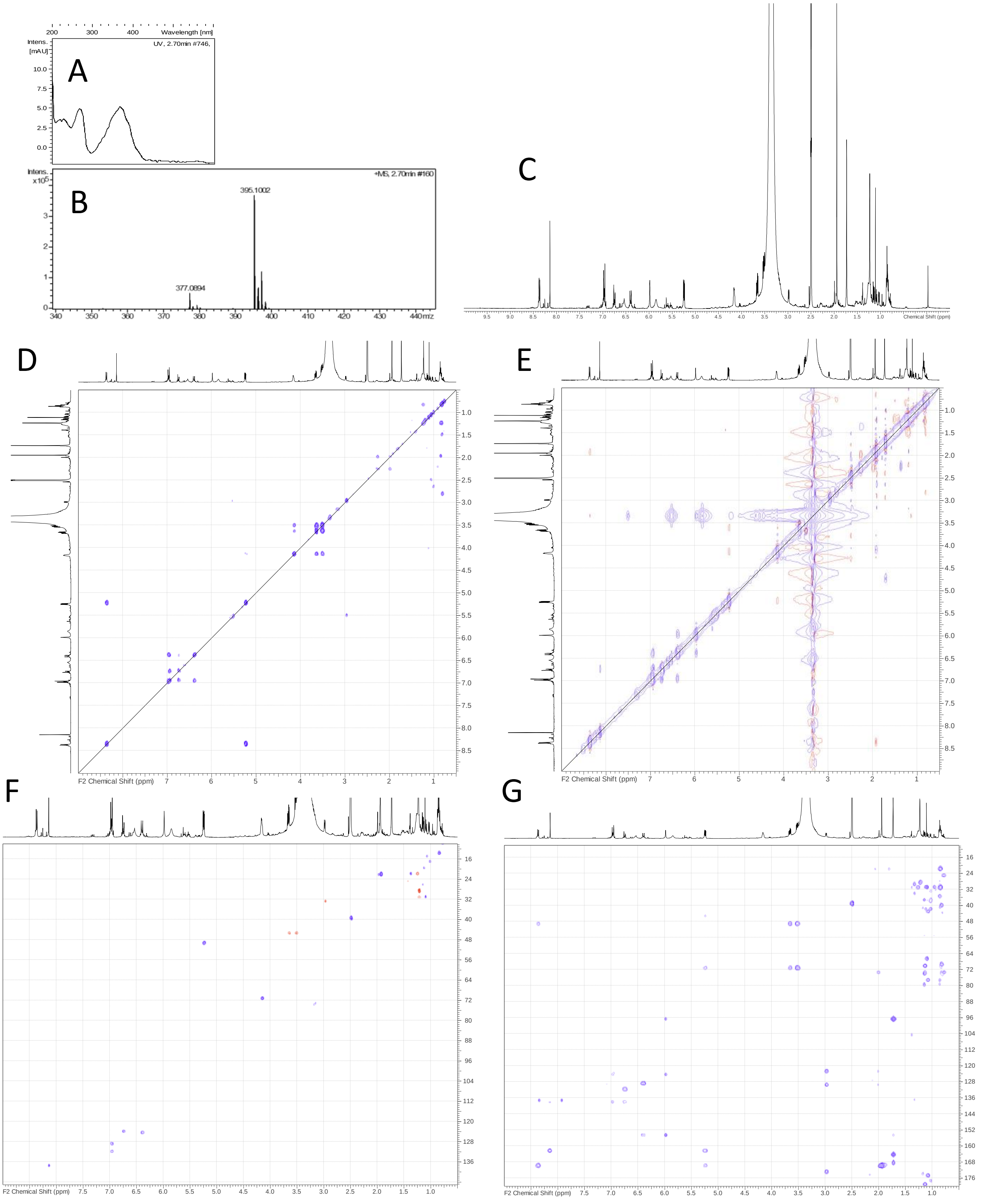

Figure S11. Spectroscopic data of LRG M3 (7): (A) UV-DAD spectrum; (B) HRMS spectrum; (C) 1H NMR spectrum in DMSO-d6, 500 MHz; (D) COSY spectrum; (E) NOESY spectrum; (F) Edited HSQC spectrum; (G) HMBC spectrum

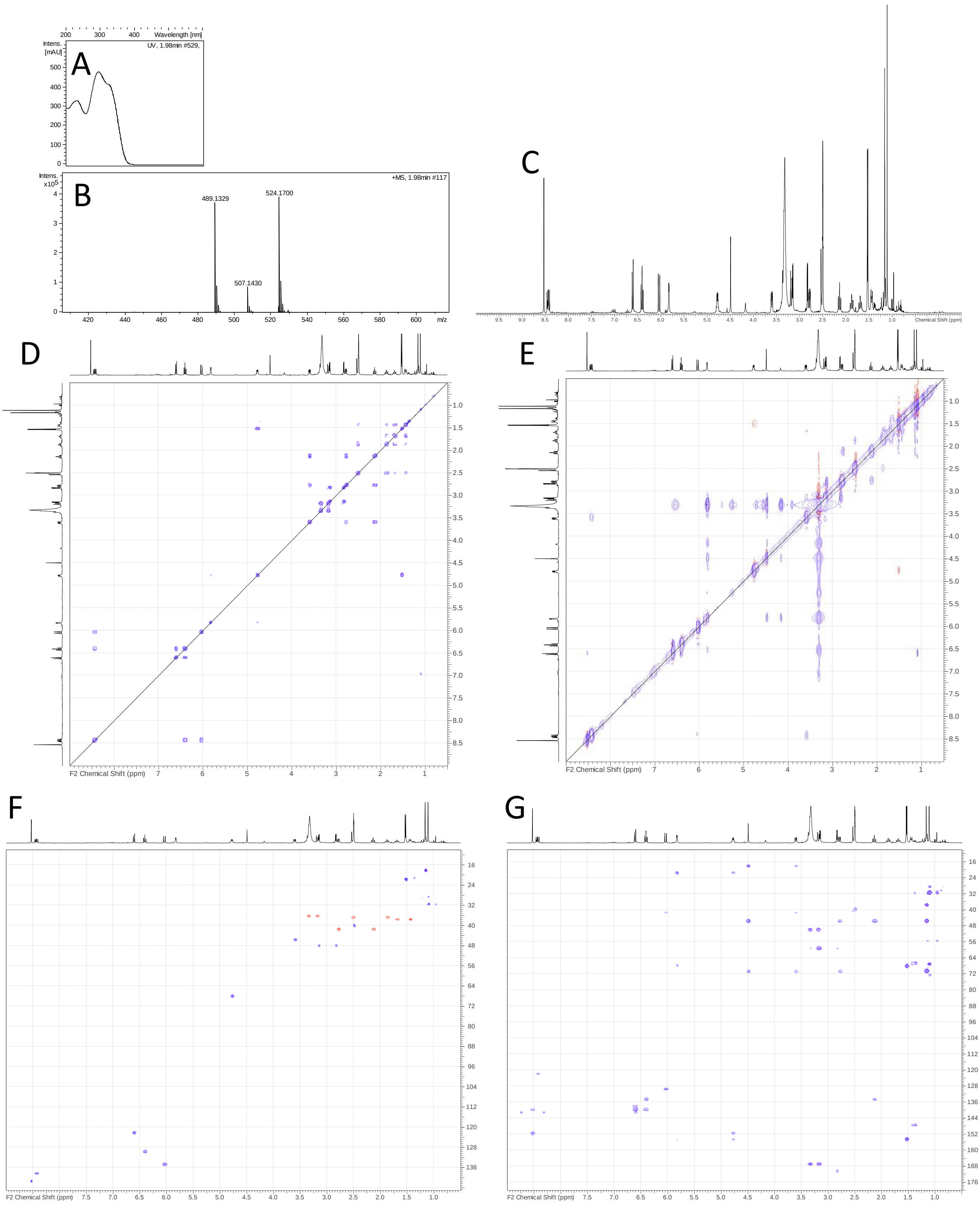

Figure S12. Spectroscopic data of LRG H1 (8): (A) UV-DAD spectrum; (B) HRMS spectrum; (C) 1H NMR spectrum in DMSO-d6, 500 MHz; (D) COSY spectrum; (E) NOESY spectrum; (F) Edited HSQC spectrum; (G) HMBC spectrum

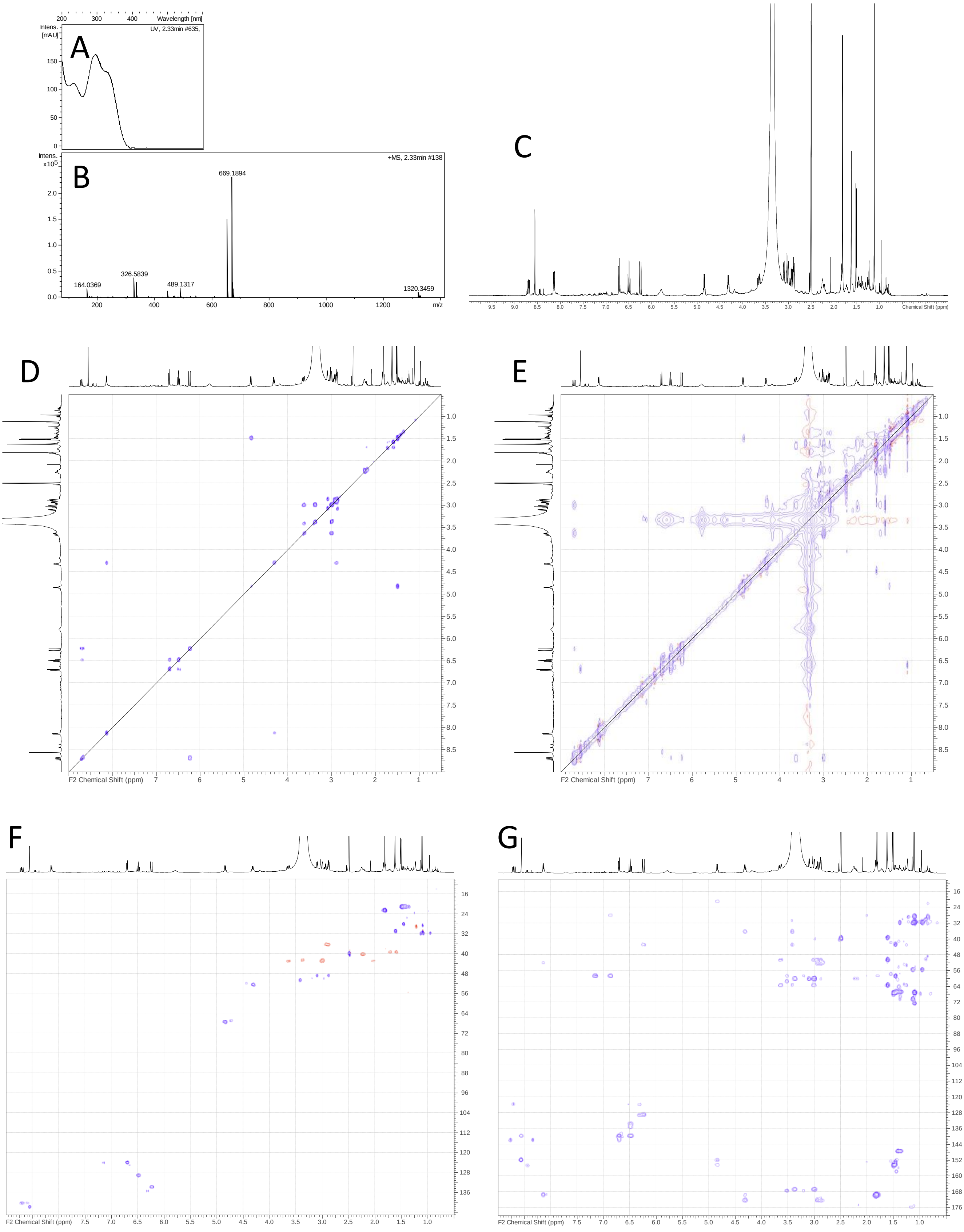

Figure S13. Spectroscopic data of LRG H2 (9): (A) UV-DAD spectrum; (B) HRMS spectrum; (C) 1H NMR spectrum in DMSO-d6, 500 MHz; (D) COSY spectrum; (E) NOESY spectrum; (F) Edited HSQC spectrum; (G) HMBC spectrum

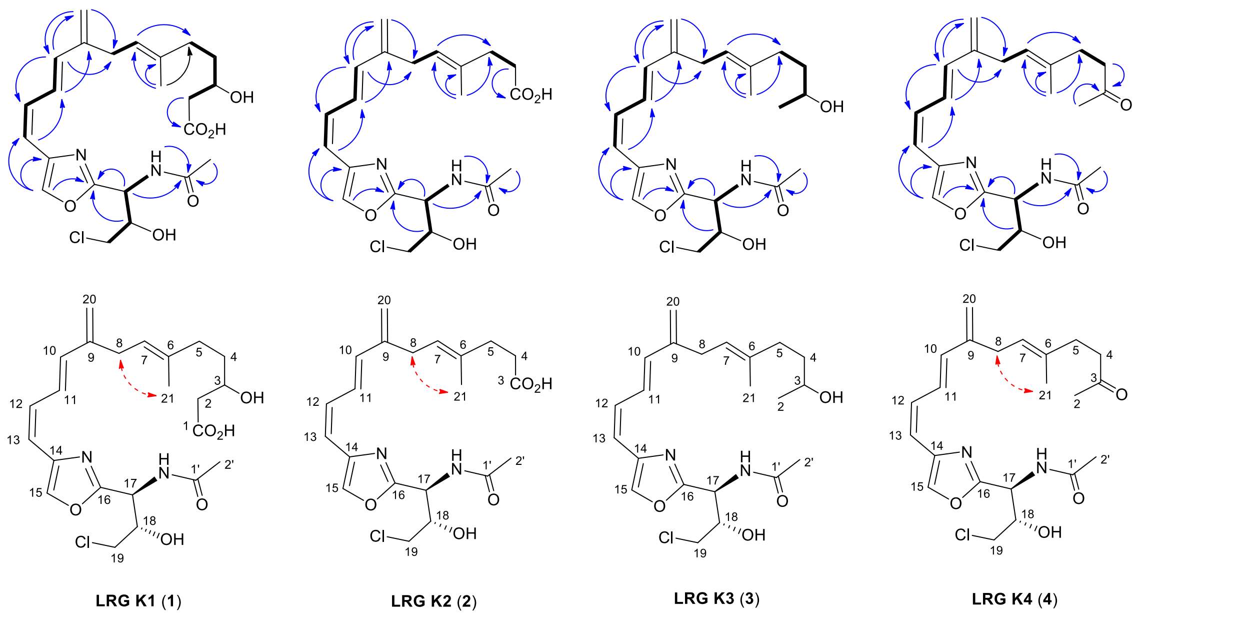

Figure S14. Key COSY correlations (bold bonds) and ^{1}H to ^{13}C HMBC correlations (blue arrows) determining the connectivity of LRG K1 (**1**), K2 (**2**), K3 (**3**) and K4 (**4**). Key NOEs (red arrows) employed alongside $^{3}J_{HH}$ and chemical shift comparisons to determine the *Z*/*E* stereochemistry of the double bonds. Absolute configuration at positions 17 and 18 maches that of L-Thr based on the biosynthesis pathway of LRGs.

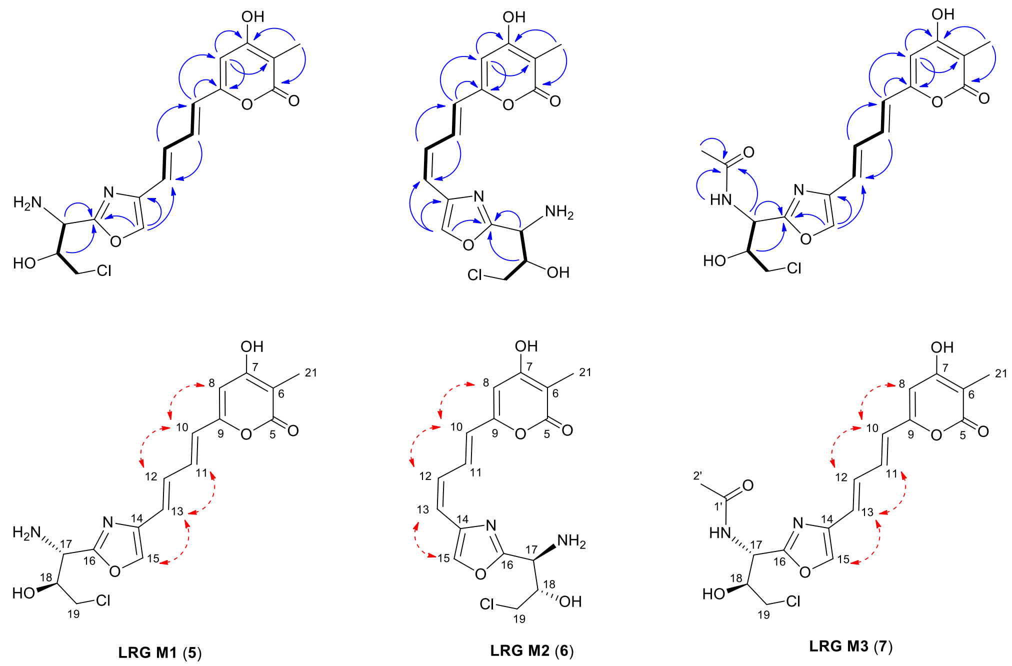

Figure S15. Key COSY correlations (bold bonds) and ^{1}H to ^{13}C HMBC correlations (blue arrows) determining the connectivity of LRG M1 (**5**), M2 (**6**), and M3 (**7**). Key NOEs (red arrows) employed alongside $^{3}J_{HH}$ and chemical shift comparisons to determine the *Z*/*E* stereochemistry of the double bonds. Absolute configuration at positions 17 and 18 maches that of L-Thr based on the biosynthesis pathway of LRGs.

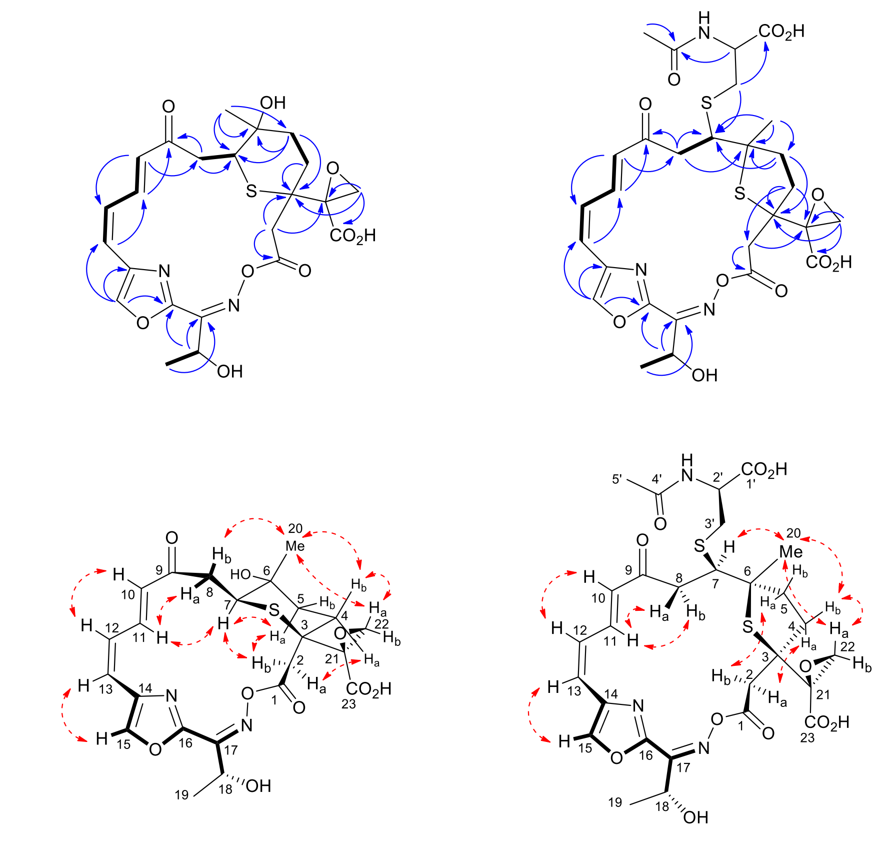

LRG H1 (8) **LRG H2 (9)**

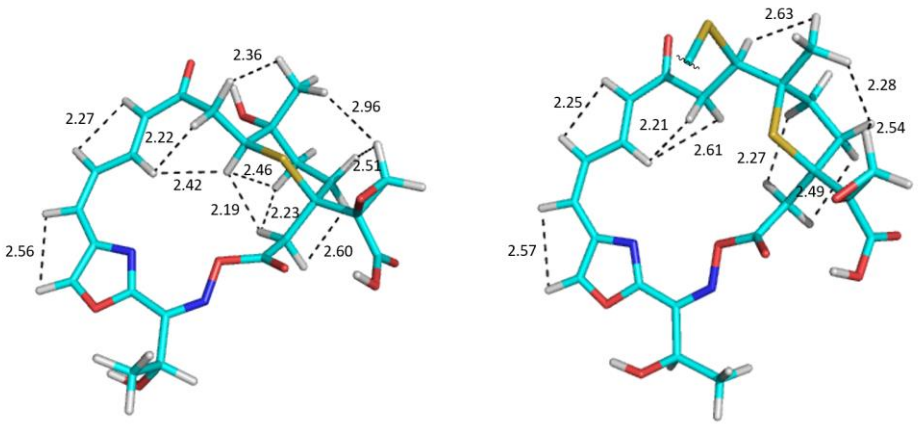

Figure S16. Key COSY correlations (bold bonds) and ^{1}H to ^{13}C HMBC correlations (blue arrows) determining the connectivity of LRG H1 (**8**) and H2 (**9**). Key NOEs (red arrows) employed alongside $^{3}J_{HH}$ and chemical shift comparisons to determine the *Z*/*E* stereochemistry of the double bonds and the relative configuration of the chiral centers. Absolute configuration at positions 17 and 18 matches that of L-Thr based on the biosynthetic pathway of LRGs. Absolute configuration at position 3 is based on common biosynthetic origin as already reported for known LRGs. Energy-minimized molecular models of both compounds are shown indicating the measured distances (in Å) related to the observed key NOEs.

Table S1: oligonucleotides used for PCR

PRIMER	SEQUENCE 5'-3'	PRIMER	SEQUENCE 5'-3'
**PRIMERS DESIGNED TO GENERATE MUTANTS**			
**Mut.orf39-41I up**	ATAGAATTCTTCCCGACCCTGCTGATG	**orf31LnmF I2 up**	CCCAAGCTTTCGATCTGATCAACAACG
**Mut.orf39-41I rp**	AAAAAGCTTACATCCCGGGTTTGACG	**orf31LnmF I2 rp**	ATACTGCAGTCGAGGGTGACGGTGAGG
**Mut.orf39-41D up**	AAAGATATCAGGACCGCAAGATCGAGG	**orf31LnmF D2 up**	TAAGATATCTGGGAGGCGGGCTCATGA
**Mut.orf39-41D rp**	CTGTCTAGAGTTGAGCCGGGACACCTC	**orf31LnmF D2 rp**	CCATCTAGAGCAGCTTGGCGTCGATC
**orf30HMG apra I up**	TAAAGATCTCTCGTTCGCGCTGCTGAT	**Hal.SyrB2 I up**	AAATCTAGACTGGGAGCGGATGTTCGTC
**orf30HMG apra I rp**	TAAAAGCTTCCCGACATAGGCGTTGG	**Hal. SyrB2 I rp**	TATGGATCCACGGTGGTGCTCCTCTCT
**orf30HMG apra D up**	AAAGATATCAACTTCCACCGGCGCTATC	**Hal.SyrB2 D up**	AACAAGCTTTACGACCACAACCGGATG
**orf30HMG apra D rp**	AAATCTAGAGTGCAGCAGGTTCATCCCG	**Hal.SyrB2 D rp**	TCGAGATCTGTGTCCAGGCTGAGGTG
**PRIMERS DESIGNED TO EXPRESS GENES**			
**ermE39-41 up**	ACAACTAGTCCTCTCCGAGCACGACACC	**ermEorf31LnmF up**	ACAACTAGTGCTGGACCGGATCGAGAA
**ermE39-41 rp**	CGCGCTAGCTCCTAGACCCACTCGTAG	**ermEorf31LnmF rp**	ATAGCTAGCGCCGGGGAAGAGCCATG
**ermEorf30HMG up**	TATACTAGTGCCTCCCAGGTCGTCCCC	**ermEHalSyrB2 up**	TAAACTAGTAGCTGTCCGCCTGCGTGG
**ermEorf30HMG rp**	TAAGCTAGCCGTGCCCAGGTCCATCT	**ermEHalSyrB2 rp**	ATAGCTAGCTCGGTGTCAGCGGGTGGT
**ermEorf29LnmE up**	ACAACTAGTGCTGCGCGCTCTTAACGT		
**PRIMERS DESIGNED TO VERIFIED MUTANTS**			
**Mut.orf39-41c up**	TCCTCACCGCGTTCCTCT	**ermEorf31LnmF up**	ACAACTAGTGCTGGACCGGATCGAGAA
**Mut.orf39-41c rp**	CGAGGGTGACGGTGGAGG	**ermEorf31LnmF rp**	ATAGCTAGCGCCGGGGAAGAGCCATG
**orf30HMG apra c2 up**	ACGATGACCGACACCCTC	**Hal.SyrB2 c up**	GCAGGACCCGAAGTGAGT
**orf30HMG apra c2 rp**	AGAGTCGGATCGTGCGGT	**Hal. SyrB2 c rp**	CTCGGTCATCGGCTCGTA

Table S2. ^{1}H **NMR (500 MHz) and** ^{13}C **NMR (125 MHz) data of LRG K1 in DMSO-**d_6**.**[a]

LRG K1		
**Position**	**δ_C, type**	**δ_H (*J* in Hz)**
1	173.0, C	
2	42.6, CH_2	a. 2.32, dd (14.7, 5.0)<br>b. 2.23, d (14.7, 8.0)
3	66.9, CH	3.79, m
4	35.4, CH_2	a. 1.50, m<br>b. 1.43, m
5	35.2, CH_2	a. 2.11, m<br>b. 2.01, m
6	136.6, C	
7	120.8, CH	5.24, m
8	29.9, CH_2	a. 2.98, dd (16.5, 7.4)<br>b. 2.93, dd (16.3, 6.7)
9	145.0, C	
10	137.0, CH	6.44, d (15.5)
11	126.5, CH	7.61, dd (15.5, 11.2)
12	130.0, CH	6.23, t (11.3)
13	117.2, CH	6.15, d (11.4)
14	138.3, C	
15	138.1, CH	8.13, s
16	162.1, C	
17	49.6, CH	5.25, m
17-NH		8.37, d (8.7)
18	71.5, CH	4.19, m
19	45.7, CH_2	a. 3.69, dd (11.1, 5.7)<br>b. 3.57, dd (11.1, 6.5)
20	116.8, CH_2	a. 5.12, br s<br>b. 5.04, br s
21	16.0, CH_3	1.62, s
1’	169.9, C	
2’	22.3, CH	1.93, s

[a] ^{13}C chemical shifts determined from the indirect dimension of HSQC and HMBC spectra

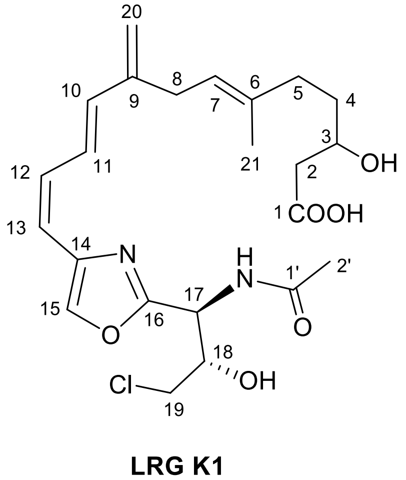

LRG K1

Table S3. ^{1}H NMR (500 MHz) and ^{13}C NMR (125 MHz) data of LRG K2 in DMSO-d_6.[a]

LRG K2		
**Position**	***δ*C, type**	***δ*H (*J* in Hz)**
3	174.1, C	
4	32.6, CH_2	2.24, br t (7.1)
5	34.2, CH_2	2.31, br t (7.1)
6	136.6, C	
7	121.3, CH	5.25, dd (8.6, 3.7)
8	29.9, CH_2	a. 2.98, dd (16.5, 7.4)<br>b. 2.93, dd (16.3, 6.7)
9	145.0, C	
10	137.0, CH	6.44, d (15.5)
11	126.5, CH	7.61, dd (15.5, 11.2)
12	130.0, CH	6.23, t (11.3)
13	117.2, CH	6.15, d (11.4)
14	138.3, C	
15	138.1, CH	8.13, s
16	162.1, C	
17	49.6, CH	5.25, m
17-NH		8.36, d (8.7)
18	71.5, CH	4.19, m
19	45.7, CH_2	a. 3.69, dd (11.1, 5.7)<br>b. 3.57, dd (11.1, 6.5)
20	116.8, CH_2	a. 5.12, br s<br>b. 5.04, br s
21	16.0, CH_3	1.62, s
1'	169.9, C	
2'	22.3, CH	1.93, s

[a] ^{13}C chemical shifts determined from the indirect dimension of HSQC and HMBC spectra

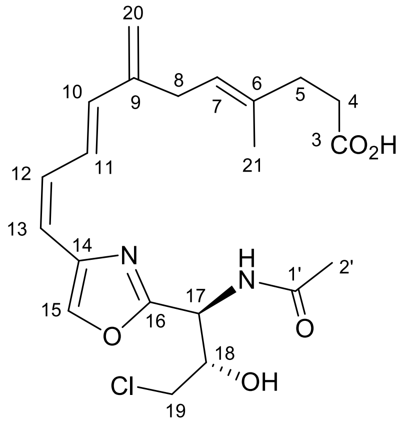

LRG K2

Table S4. ^{1}H NMR (500 MHz) and ^{13}C NMR (125 MHz) data of LRG K3 in DMSO-d_6.[a]

LRG K3		
**Position**	**δ_C, type**	**δ_H (*J* in Hz)**
2	23.5, CH_3	1.03, d (6.2)
3	65.6, CH	3.55, m
4	37.5 CH_2	1.41, m
5	35.4, CH_2	2.00, m
6	136.6, C	
7	120.8, CH	5.23, m
8	29.9, CH_2	2.96, m
9	145.0, C	
10	137.0, CH	6.43, d (15.5)
11	126.5, CH	7.59, dd (15.5, 11.2)
12	130.0, CH	6.23, t (11.3)
13	117.2, CH	6.15, d (11.4)
14	138.3, C	
15	138.1, CH	8.13, s
16	162.1, C	
17	49.6, CH	5.25, m
17-NH		8.36, d (8.6)
18	71.5, CH	4.19, m
19	45.7, CH_2	a. 3.69, m<br>b. 3.57, m
20	116.8, CH_2	a. 5.12, br s<br>b. 5.03, br s
21	16.0, CH_3	1.62, s
1'	169.9, C	
2'	22.3, CH	1.93, s

[a] ^{13}C chemical shifts determined from the indirect dimension of HSQC and HMBC spectra

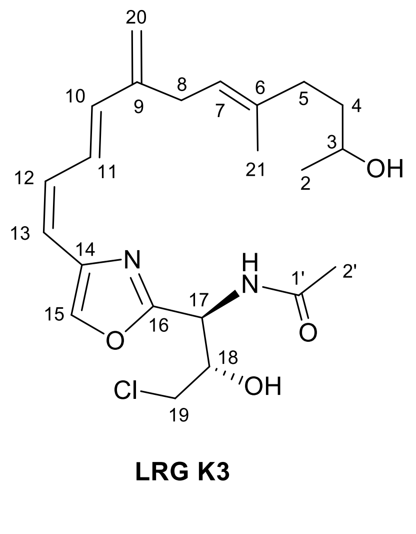

LRG K3

Table S5. ^{1}H NMR (500 MHz) and ^{13}C NMR (125 MHz) data of LRG K4 in DMSO-d_6.[a]

	LRG K4	
**Position**	**δ_C, type**	**δ_H (*J* in Hz)**
2	29.6, CH_3	2.06, s
3	208.0, C	
4	41.4, CH_2	2.52, m
5	33.1, CH_2	2.19, br t (7.1)
6	136.6, C	
7	121.3, CH	5.22, dd (8.6, 3.7)
8	29.9, CH_2	a. 2.98, dd (16.5, 7.4)<br>b. 2.93, dd (16.3, 6.7)
9	145.0, C	
10	137.0, CH	6.43, d (15.5)
11	126.5, CH	7.59, dd (15.5, 11.2)
12	130.0, CH	6.23, t (11.3)
13	117.2, CH	6.15, d (11.4)
14	138.3, C	
15	138.1, CH	8.13, s
16	162.1, C	
17	49.6, CH	5.25, m
17-NH		8.36, d (8.7)
18	71.5, CH	4.19, m
19	45.7, CH_2	a. 3.69, dd (11.1, 5.7)<br>b. 3.57, dd (11.1, 6.5)
20	116.8, CH_2	a. 5.11, br s<br>b. 5.02, br s
21	16.0, CH_3	1.62, s
1'	169.9, C	
2'	22.3, CH	1.93, s

[a] ^{13}C chemical shifts determined from the indirect dimension of HSQC and HMBC spectra

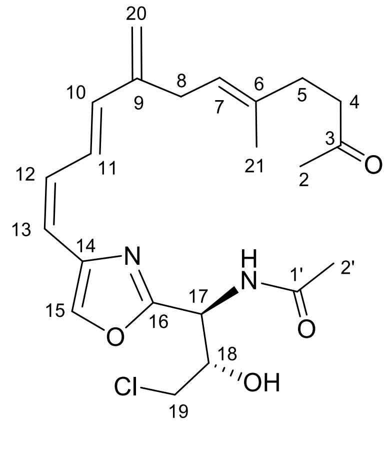

LRG K4

Table S6. ^{1}H NMR (500 MHz) and ^{13}C NMR (125 MHz) data of LRG M1 in DMSO-d$_6$.[a]

LRG M1		
Position	δ_C, type	δ_H (J in Hz)
5	164.4, C	
6	99.1, C	
7	164.6, C	
8	101.7, CH	6.22, s
9	155.5, C	
10	124.0, CH	6.47, d (14.7)
11	132.9, CH	7.07, dd (14.6, 11.3)
12	129.3, CH	7.01, dd (14.5, 11.3)
13	124.5, CH	6.85, d (14.5)
14	138.6, C	
15	138.7, CH	8.34, s
16	159.3, C	
17	50.4, CH	4.58, d (5.7)
18	70.1, CH	4.22, br q (5.6)
19	45.6, CH_2	a. 3.79, dd (11.4, 4.8)<br>b. 3.61, dd (11.4, 5.9)
21	8.8, CH_3	1.80, s

[a] ^{13}C chemical shifts determined from the indirect dimension of HSQC and HMBC spectra

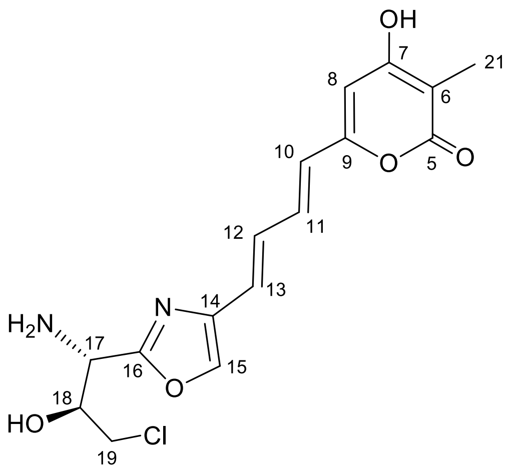

LRG M1

Table S7. ^{1}H NMR (500 MHz) and ^{13}C NMR (125 MHz) data of LRG M2 in DMSO-d$_6$.[a]

LRG M2		
**Position**	**δ_C, type**	**δ_H (*J* in Hz)**
5	164.5, C	
6	99.3, C	
7	164.8, C	
8	102.2, CH	6.27, s
9	155.5, C	
10	125.9, CH	6.46, d (15.4)
11	130.5, CH	8.05, m
12	129.2, CH	6.40, t (11.4)
13	120.3, CH	6.42, d (11.1)
14	138.3, C	
15	140.3, CH	8.41, s
16	158.0, C	
17	50.2, CH	4.64, d (6.7)
18	69.6, CH	4.32, br q (5.4)
19	46.0, CH_2	a. 3.85, dd (11.8, 4.4)<br>b. 3.72, dd (11.8, 5.2)
21	8.8, CH_3	1.80, s

[a] ^{13}C chemical shifts determined from the indirect dimension of HSQC and HMBC spectra

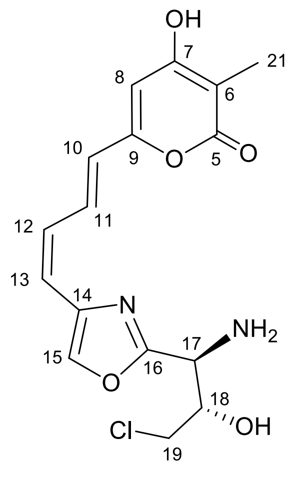

LRG M2

Table S8. ^{1}H NMR (500 MHz) and ^{13}C NMR (125 MHz) data of LRG M3 in DMSO-d$_6$.[a]

LRG M3		
Position	δ_C, type	δ_H (*J* in Hz)
5	168.5, C	
6	97.0, C	
7	164.4, C	
8	104.4, CH	5.98, s
9	154.8, C	
10	124.5, CH	6.40, d (14.5)
11	131.9, CH	6.98, dd (14.5, 10.8)
12	128.9, CH	6.95, dd (14.5, 10.8)
13	124.1, CH	6.75, d (14.2)
14	138.4, C	
15	137.5, CH	8.14, s
16	162.6, C	
17	49.5, CH	5.24, dd (8.7, 3.7)
17-NH		8.37, d (8.7)
18	71.4, CH	4.16, m
19	45.6, CH_2	a. 3.66, dd (11.1, 5.7)<br>b. 3.52, dd (11.1, 6.5)
21	9.1, CH_3	1.73, s
1'	22.3, CH_3	1.94, s
2'	169.9, C	

[a] ^{13}C chemical shifts determined from the indirect dimension of HSQC and HMBC spectra

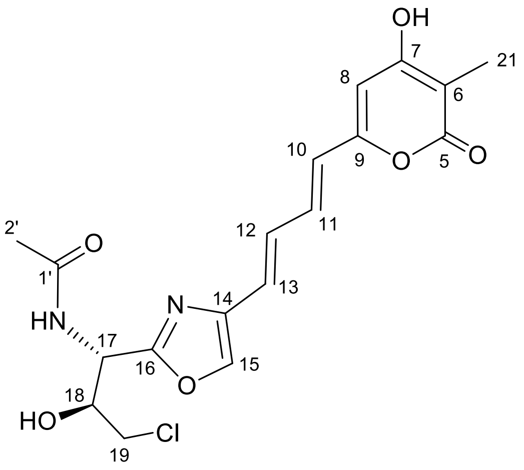

LRG M3

Table S9. ^{1}H NMR (500 MHz) and ^{13}C NMR (125 MHz) data of LRG H1 in DMSO-d_6.[a]

	LRG H1	
Position	δ_C, type	δ_H (*J* in Hz)
1	167.2, C	
2	36.5, CH_2	3.35, d (14.2)<br>3.18, d (14.2)
3	50.3, C	
4	37.1, CH_2	a. 2.52, m<br>b. 1.88, td (14.2, 3.1)
5	37.9, CH_2	a. 1.69, td (14.2, 2.5)<br>b. 1.44, dt (14.2, 3.4)
6	71.1, C	
7	45.9, CH	3.60, dd (12.8, 4.0)
8	41.8, CH_2	a. 2.79, dd (13.0, 4.0)<br>b. 2.14, t (13.0)
9	198.3, C	
10	134.8, CH	6.04, d (16.2)
11	138.4, CH	8.43, dd (16.2, 11.4)
12	129.7, CH	6.41, t (11.5)
13	122.2, CH	6.60, d (11.5)
14	139.9, C	
15	141.5, CH	8.53, s
16	151.9, C	
17	154.9, C	
18	68.2, CH	4.78, q (6.7)
19	21.9, CH_3	1.53, d (6.7)
20	18.5, CH_3	1.16, s
21	59.6, C	
22	48.3, CH_2	a. 3.15, d (5.8)<br>b. 2.83, d (5.8)
23	170.7, C	

[a] ^{13}C chemical shifts determined from the indirect dimension of HSQC and HMBC spectra

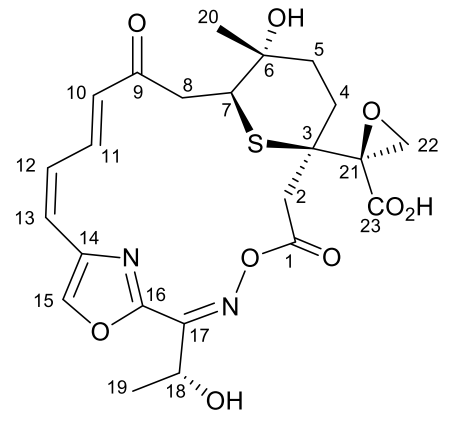

LRG H1

Table S10. ^{1}H NMR (500 MHz) and ^{13}C NMR (125 MHz) data of LRG H2 in DMSO-d_6.[a]

LRG H2		
Position	δ_C, type	δ_H (J in Hz)
1	167.0, C	
2	42.9, CH_2	3.37, m<br>3.00, m
3	60.4, C	
4	40.4, CH_2	a. 2.26, m<br>b. 2.22, m
5	39.7, CH_2	a. 1.73, m<br>b. 1.61, m
6	63.7, C	
7	50.9, CH	3.43, m
8	43.2, CH_2	a. 3.65, dd (18.4 6.6)<br>b. 3.01, m
9	198.3, C	
10	134.0, CH	6.25, d (16.1)
11	140.3, CH	8.70, dd (16.1, 11.4)
12	129.3, CH	6.49, t (11.4)
13	124.0, CH	6.71, d (11.4)
14	139.8, C	
15	141.9, CH	8.56, s
16	152.1, C	
17	154.6, C	
18	67.7, CH	4.84, q (6.7)
19	21.5, CH_3	1.51, d (6.7)
20	31.1, CH_3	1.62, s
21	60.4, C	
22	49.1, CH_2	a. 3.10, d (5.8)<br>b. 2.89, m
23	171.3, C	
1’	172.4, C	
2’	52.8, CH	4.32, ddd (13.1, 7.8, 5.4)
2’-NH		8.14, d (7.9)
3’	36.6, CH_2	a. 2.95, dd (13.1, 5.4)<br>b. 2.89, m
4’	169.7, C	
5’	22.9, CH_3	1.81, s

[a] ^{13}C chemical shifts determined from the indirect dimension of HSQC and HMBC spectra

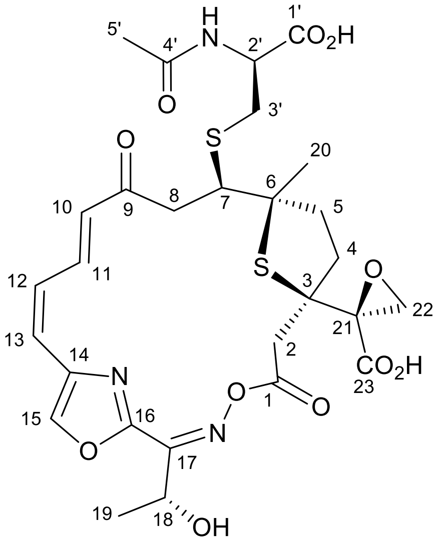

LRG H2
